# Development of Bovine Gastric Organoids as a Novel *In Vitro* Model to Study Host-Parasite Interactions in Gastrointestinal Nematode Infections

**DOI:** 10.3389/fcimb.2022.904606

**Published:** 2022-06-30

**Authors:** Marc N. Faber, David Smith, Daniel R. G. Price, Philip Steele, Katie A. Hildersley, Liam J. Morrison, Neil A. Mabbott, Alasdair J. Nisbet, Tom N. McNeilly

**Affiliations:** ^1^ Moredun Research Institute, Pentlands Science Park, Penicuik, United Kingdom; ^2^ Roslin Institute, Royal (Dick) School of Veterinary Studies, University of Edinburgh, Penicuik, United Kingdom

**Keywords:** three-dimensional (3D) organoids, host-pathogen interactions, gastrointestinal, *Ostertagia ostertagi*, tissue remodelling, nematodes

## Abstract

Gastro-intestinal nematode (GIN) parasites are a major cause of production losses in grazing cattle, primarily through reduced growth rates in young animals. Control of these parasites relies heavily on anthelmintic drugs; however, with growing reports of resistance to currently available anthelmintics, alternative methods of control are required. A major hurdle in this work has been the lack of physiologically relevant *in vitro* infection models that has made studying precise interactions between the host and the GINs difficult. Such mechanistic insights into the infection process will be valuable for the development of novel targets for drugs, vaccines, or other interventions. Here we created bovine gastric epithelial organoids from abomasal gastric tissue and studied their application as *in vitro* models for understanding host invasion by GIN parasites. Transcriptomic analysis of gastric organoids across multiple passages and the corresponding abomasal tissue showed conserved expression of tissue-specific genes across samples, demonstrating that the organoids are representative of bovine gastric tissue from which they were derived. We also show that self-renewing and self-organising three-dimensional organoids can also be serially passaged, cryopreserved, and resuscitated. Using *Ostertagia ostertagi*, the most pathogenic gastric parasite in cattle in temperate regions, we show that cattle gastric organoids are biologically relevant models for studying GIN invasion in the bovine abomasum. Within 24 h of exposure, exsheathed larvae rapidly and repeatedly infiltrated the lumen of the organoids. Prior to invasion by the parasites, the abomasal organoids rapidly expanded, developing a ‘ballooning’ phenotype. Ballooning of the organoids could also be induced in response to exposure to parasite excretory/secretory products. In summary, we demonstrate the power of using abomasal organoids as a physiologically relevant *in vitro* model system to study interactions of *O. ostertagi* and other GIN with bovine gastrointestinal epithelium.

## Introduction

Gastro intestinal nematodes (GIN) infect millions of cattle globally and represent a major constraint to efficient livestock production (Charlier et al., 2015a).While clinical disease due to GIN is rare due to extensive use of anthelmintic treatments, sub-clinical infections in cattle are common and cause reductions in live-weight gain, milk production, and carcass quality (Charlier et al., 2014). Efforts to estimate the cost of GIN infections to the cattle industry have focused on gains in productivity following anthelmintic treatment rather than exact monetary costs (Charlier et al., 2015b). These studies reveal average differences in weight gain between treated and untreated first grazing season calves of between 150-315g/day (Shaw et al., 1998), and increases in milk production of around 0.35-1 kg per cow per day following treatment (Sanchez et al., 2004). *Ostertagia ostertagi* is the most economically important GIN in cattle in temperate areas, largely because immunity to reinfection by the parasite is slow to develop and often incomplete even after months of exposure (Klesius, 1993; Tuo et al., 2021). The problem of *O. ostertagi* infection is compounded by recent reports of widespread resistance to all three major classes of anthelmintic drug currently used to control the parasite (Geurden et al., 2015; Kelleher et al., 2020; Rose Vineer et al., 2020; Bartley et al., 2021). Alternative methods of control, including vaccines or novel drug treatments, are therefore required.

*Ostertagia ostertagi* has a direct life-cycle with a pre-patent period of around 18-21 days; like other trichostrongylid parasites, infective third stage larvae (L3) are ingested through grazing on contaminated pasture and penetrate the gastric glands in the abomasum (gastric stomach) where they develop into fourth stage larvae (L4) before emerging into the lumen at around 10-14 days to become sexually mature adults (Klesius, 1993; Fox, 1997). Infection with this parasite causes significant pathology, particularly when L4s emerge from the gastric gland. This can cause hyperplasia of the gastric glands, epithelial cytolysis, and loss of parietal cells, resulting in elevated abomasal pH and impaired protein metabolism (Fox, 1997). Key early events in the infection process are associated with profound changes to the gastric epithelium, including dilation of parasitised gastric glands and the de-differentiation of the epithelium within these glands (Murray et al., 1970; Snider et al., 1988). However, the mechanisms by which *O. ostertagi* invades gastric glands and modulates gastric epithelial function are poorly understood. Addressing these important knowledge gaps is hindered due to the inaccessibility of the abomasum for the *in vivo* temporal analysis of early host-pathogen interactions, as well as the lack of physiologically-relevant *in vitro* systems in which to study these events.

Several pioneering studies have used organoids or “mini-guts” to study interactions between GIN and the gastro-intestinal (GI) epithelium. These organoids are derived from tissue resident, self-renewing intestinal adult stem cells, and consist of organ-specific tissue (gastric or intestinal epithelia) which self-organise into three-dimensional structures with a single epithelial layer and central lumen (Sato et al., 2009; Barker et al., 2010). Importantly, GI organoids are highly representative of the *in vivo* epithelial environment as they contain a range of differentiated cell types which spatially organise into gastric gland-like structures. Studies have shown how murine intestinal organoids can be used to investigate the effects of parasite extracts or excretory-secretory products (ESP), including extracellular vesicles (Eichenberger et al., 2018b; Luo et al., 2019; Drurey et al., 2022). However, we have shown that the infectious L3 larval forms of *Teladorsagia circumcincta*, a highly pathogenic ovine gastric parasite closely related to *O. ostertagi*, specifically invade ovine gastric organoids and reside within the lumen in a manner analogous to the invasion of gastric glands *in vivo* (Smith et al., 2021). This indicates that in addition to defining the effects of specific parasite-derived molecules on epithelial cell function, organoid cultures may also represent a physiologically relevant system to study physical parasite invasion processes.

In this study, we have built upon our work in sheep by developing and characterising bovine abomasal organoids, and used these organoids to define early interactions between *O. ostertagi* L3 and the abomasal epithelium. Here we report the first detailed analysis to date of live parasite-organoid interactions in livestock. We demonstrate that *O. ostertagi* L3 specifically invade bovine abomasal organoids and release heat-labile molecules which rapidly expand organoids prior to parasite invasion. The organoid culture system developed in this study represents a disruptive technology that has the potential to identify novel GIN molecules involved in gastric gland invasion. Such molecules may represent useful targets for drug and/or vaccine interventions aimed at controlling *O. ostertagi* infections in cattle.

## Materials and Methods

### Animals

All animal procedures were performed at Moredun Research Institute (MRI), UK under license as required by the UK Animals (Scientific Procedures) Act 1986, with ethical approval from the MRI Animal Experiments Committee. Animals were raised at MRI under conditions designed to exclude accidental infection with helminth parasites and were confirmed helminth-naïve. The bovine abomasum tissue used to generate bovine abomasal organoids in this study were derived from uninfected 4-month-old male Aberdeen Angus (Calf 1, C1) and male Holstein-Friesian calves (C2, C3). Abomasal tissue for histology was collected from Holstein-Friesian calves infected with 50,000 *O. ostertagi* L3 larvae (MOo2 strain) at day 21 post-infection.

### Isolation of Gastric Glands

The isolation of gastric glands was performed as described previously (Smith et al., 2021). In short, calf abomasum was removed post-mortem and opened from the omasum-abomasal opening to the pyloric sphincter. Upon opening, the abomasal contents were removed by rinsing with tap water. A 10 cm^2^ fold from the abomasal fundus was sampled using a sterile scalpel and forceps and placed in sterile ice-cold Hank’s buffered saline solution containing 25 µg/ml gentamicin (G1397-10ML; Sigma-Aldrich, USA) and 100 U/ml penicillin/streptomycin (abHBSS) and kept on ice until processing. The abomasal folds were placed into sterile Petri dishes and, using sterile glass slides, the mucus layer was removed by gentle scraping. Epithelial tissue was then scraped off using glass slides and collected into a sterile Falcon tube containing abHBSS. Samples were centrifuged at 400 x *g* for 2 min to gently pellet the epithelial tissue. After removing the supernatant, the residual top mucus layer was removed with a stripette and the pellet was resuspended in abHBSS and centrifuged again. This process was repeated until the mucus layer was no longer visible following centrifugation. Pellets were resuspended in 25 ml of digestion medium (Dulbecco’s Modified Eagle Medium [DMEM] high glucose, (11574486; Gibco) 1% FBS, 20 µg/ml dispase (4942086001; Roche), 75 U/ml collagenase (C2674; Sigma-Aldrich), 25 µg/ml gentamicin (G1272-10ML, Merck) and 100 U/ml penicillin/streptomycin) and incubated horizontally in a shaking incubator at 80 rpm for 40 minutes at 37°C. The tube was gently shaken and after the undigested material settled, the supernatant was transferred into a new 50ml Falcon tube which was then centrifuged at 400 x *g* for 2 minutes. Following centrifugation, the supernatant was discarded, and the pellet resuspended in 50 ml abHBSS. Samples were then centrifuged again at 400 x *g* for 2 minutes. The final pellet was resuspended in 3 ml advanced DMEM/F12 (12634-010; Gibco) containing 1X B27 supplement minus vitamin A (12587-010; Gibco), 25 µg/ml gentamicin and 100 U/ml penicillin/streptomycin (advDMEM).

### Organoid Culture, Passaging, and Cryopreservation

Abomasal crypts were diluted to a density of 400 crypts/100 µl in advDMEM and mixed with 150 µl of BD Growth Factor Reduced Matrigel™ Matrix (356230; BD Biosciences) on ice. Fifty microliter droplets were added to consecutive wells of a 24-well tissue culture plate (3524, Corning) that had been pre-warmed to 37°C and incubated at 37°C, 5% CO_2_ for 10 minutes to allow the Matrigel™ to polymerize. 550 µl of complete IntestiCult Growth Medium (mouse) (6005; STEMCELL Technologies) containing 500 nM Y-27632 (10005583; Cambridge Bioscience), 10 µM LY2157299 (15312; Cambridge Bioscience), 10 µM SB202190 (ALX-270-268-M001; Enzo Life Sciences) and gentamicin (50 µg/ml) was added to each well containing a Matrigel™ dome and incubated at 37°C, 5% CO_2._ IntestiCult Growth Medium was changed every 2-3 days and organoids were cultured for 7-14 days prior to passaging, at which stage they were deemed mature, having a spherical morphology and a clearly distinguishable lumen. To passage abomasal organoids, media was removed and 1ml of ice-cold advDMEM/F12 was added to each well to depolymerise the Matrigel™. Resuspended organoids were pooled from 4 wells and transferred into a 15 ml Falcon tube on ice for 5 minutes for the organoids to settle. The supernatant was removed and organoids resuspended in 300 µl advDMEM/F12. Organoids were then mechanically disrupted by repeated pipetting (150-200 passes through the pipette tip), using a bent 200 µl pipette tip to increase shearing force. Organoid fragments were counted and diluted to 400-1000 crypts per 100 µl and seeded as described above. For each passage, organoids were cryo-preserved by dissolving the Matrigel™ as described above using cold advDMEM/F12 and transferred into a microcentrifuge tube to be pelleted at 290 x *g* for 5 minutes at 4°C. Approximately 1,000 crypts were resuspended in a 1 ml Cryostor CS10 cryopreservation medium (STEMCELL Technologies, Canada) and transferred into a cryovial. Cryovials were stored in a cryogenic freezing container and placed at -70°C for 2-3 hours before subsequently being transferred to -196°C for long-term storage. Resuscitation of frozen organoids was performed as described previously (Smith et al., 2021). Phase contrast microscopy was used to image organoids from passage zero to two and following nine days of *in vitro* growth at passage two. In addition, time-lapse microscopy was performed in an environmental control chamber at 37°C, 5% CO_2_ on a Zeiss Axiovert 200M microscope using Axiovision as operating software.

### RNA Extraction of Bovine Abomasum Tissue and Abomasum Organoids

Animal tissue samples for RNA extraction were sampled from RNAlater (Sigma-Aldrich) preserved sections of the same abomasal fold that was used for primary crypt extraction and organoid culture. Organoid samples for RNA extraction were generated by cultivating organoids up to passage 4 and harvesting organoids at each passage. To ensure sufficient RNA yield, four wells of organoids were pooled for RNA extraction by dissolving organoid-containing Matrigel™ domes as described above and transferring all samples into the same 15 ml sterile Falcon tube. Samples were pelleted by gentle centrifugation at 290 x *g* for 5 minutes at 4°C. and subsequently resuspended in 350 µl RLT buffer (Qiagen) containing β-mercaptoethanol, snap frozen in liquid nitrogen, and stored at -70^°^C until processing. RNA extraction was performed on organoid and tissue samples at the same time using a RNeasy mini kit (Qiagen) according to the manufacturer’s protocol, and an on-column DNase digest was performed. Approximately 30 mg of tissue samples were homogenized in 600 µl of RLT buffer containing β-mercaptoethanol using a Precellys^®^ Tissue Homogenizer with CK28 tubes (Bertin Instruments™, France) with 3 x 30s pulses at 5500 rpm and 5 min on ice between each pulse. RNA was extracted as described above and stored at -70^°^C until RNA-seq analysis. In total, RNA samples were generated from three individual cattle and from P0-P4 organoids that were derived from the abomasum tissue of those animals.

### RNA-Seq Analysis

For each tissue and organoid sample, 2 µg of total RNA was used for RNA-seq analysis. All library synthesis and sequencing were performed by Veterinary Medical Research & Development, Zoetis. In brief, libraries were prepared using the Illumina Stranded mRNA Prep as per the manufacturer’s protocol, following polyA+ tail mRNA capturing. A total of 18 libraries were constructed for primary tissue and organoid passages P0-P4 derived from three individual animals. Library quality control was performed by qPCR, as well as 4200 TapeStation (Agilent Technologies, USA) analysis. Samples were combined into 3 sub-pools and were sequenced using a NextSeq550 system (Illumina, USA) with a paired end 2x100 bp run resulting in 29.5 million reads per sample. Sequence reads were checked for quality using FastQC v0.11.7 and pseudo-aligned to the *Bos taurus* transcriptome (GCF_002263795.1) using Kallisto v0.46.2 with default settings (Bray et al., 2016), resulting in an average mapping rate of 82.3% matched reads. The transcript read abundance, calculated as transcripts per million (TPM), was used for downstream analysis, with a 100 TPM cut-off across all samples. Comparisons of the gene expression profile of each sample were performed by principal component analysis (PCA) of the top 500 expressed genes using pcaExplorer version 2.12.0 R/Bioconductor package (Marini and Binder, 2019). To uncover abomasum–enriched transcripts, previously published RNA-seq data on bovine intestinal crypts and organoids (Gene Expression Omnibus data GSE112674) were included in the analysis (Hamilton et al., 2018) and analysed in parallel to the bovine abomasum transcript dataset. Specific genes of interest were also manually retrieved and used to generate heatmaps after log_2_ transformation of TPM data using GraphPad Prism software (v8.0). Genes selected for specific abomasal cell types were based on our previous publication on ovine abomasum RNA-seq and the top ten transcripts for parietal, mucus, tuft, and enterochromaffin-like in a published ovine single-cell RNA-seq abomasum dataset (Hildersley et al., 2021; Smith et al., 2021). The mRNA-seq analysis data sets are available at the NCBI Sequence Read Archive (SRA) Database under the project accession number PRJNA806768.

### Bioinformatics Packages

For comparative analysis of Illumina sequence data, we utilised publically available software packages. Illumina reads were initially checked for sequence quality using FastQC v0.11.7 and then mapped to the *Bos taurus* transcriptome (GCF_002263795.1) using Kallisto v0.46.2 with default settings (Bray et al., 2016). The count matrix was trimmed to remove genes with read sum counts <100 TPM across samples. For comparative gene expression analysis we used the pcaExplorer version 2.12.0 R/Bioconductor package, which includes tools for data normalisation, heatmap generation, and functional interpretation of the principal components (Marini and Binder, 2019). In brief, the read count matrix was imported and normalised using the median of ratios method “estimateSizeFactors()” from the DESeq2 package, which has been shown to perform robustly in many scenarios under the assumption that most genes are not differentially expressed (Dillies et al., 2013). Data visualisations were generated within the pcaExplorer package using the base and ggplot2 graphics systems in R.

### Immunohistochemistry (IHC)

Abomasum organoids (P2) were cultivated in 40 µl Matrigel™ domes using 400 µl IntestiCult Growth Medium for a minimum of 7 days in 8-well Nunc Lab-Tek II Chamber Slide System (Fisher Scientific, USA) as described above. After removing the culture medium, organoids were fixed in ice-cold neutral buffered 10% formalin (Sigma-Aldrich) for 30 minutes at 4°C. For staining, organoids were washed twice in washing buffer (WB, 0.1% Tween20 in PBS), media removed, and then permeabilised using 0.1% TritonX-100 in PBS for 20 minutes at room temperature, followed by three washes with washing buffer. Organoids were blocked for 30 minutes in 1% BSA in WB buffer at RT after which the primary antibody was diluted in the blocking solution and incubated on the organoids overnight at 4°C. The primary antibodies used to stain for epithelial markers were polyclonal rabbit α-EPCAM (ab71916, Abcam, used at a 1:600 dilution) and monoclonal mouse α-pan cytokeratin (used at a 1:100 dilution), as well as the proliferation marker polyclonal rabbit α-Ki67 (ab15580, Abcam, 1:500 dilution). Mouse IgG1/IgG2a (in house) or rabbit IgG (Sigma-Aldrich) were used as isotype controls and were diluted at 1:100 for mouse IgG1/IgG2a and 1:5550 for rabbit IgG. The following day, samples were washed three times in WB buffer, and the secondary antibodies diluted in blocking buffer and incubated for 1 hour at RT. The Secondary antibodies used were goat α-mouse Alexa Fluor 488 (ab150117, abcam) and goat anti-rabbit Alexa Fluor 488 (ab150081, Abcam), and both were used at concentrations 1:500. After washing the samples three times with IF buffer, nuclei, and F-actin were stained with Hoechst 33258 (94403, Sigma-Aldrich, 1:200 dilution) and Phalloidin-iFluor (ab176756, Abcam, 1:1000), respectively, by incubation for 10 minutes at RT followed by three washes with IF buffer. Chamber slide well casings were removed and slides were mounted using ProLong Diamond antifade mountant (P36965, Thermo Fisher Scientific).

Organoids treated only with their respective isotype controls (mouse or rabbit serum IgG) were negative for any labelling (**Supplementary Figure 2**). Organoids were imaged using a Zeiss Axiovert 200 M microscope using an AxioCamMR3 and Zen Blue (version 3.2) as operating software.

### Organoid and Tissue Histology

*Ostertagia ostertagi*-infected abomasal tissue was fixed in neutral buffered Formalin 10% (Sigma-Aldrich) for 24 h at room temperature (RT), washed twice in PBS, and stored in 70% EtOH until processing. Fixed tissue samples were embedded in paraffin wax (PFPE), dewaxed and 3 µm sections prepared. Hematoxylin and eosin (HE) and Periodic acid–Schiff (PAS) staining were performed as described previously (Albuquerque et al., 2019; Hildersley et al., 2021). To generate sections of organoids, Matrigel™ domes containing organoids were embedded in Epredia HistoGel Specimen Processing Gel (Thermo Fisher Scientific) as described previously (Pinto et al., 2011). Briefly, the medium was removed from day 7-9 organoids grown in Nunc Lab-Tek II Chamber Slides (Fisher Scientific), and chamber slide well cassettes removed. Matrigel™ domes were carefully removed using a scalpel blade and placed into 9 mm^2^ histology moulds that had been pre-filled with 100µl HistoGel. Three Matrigel™ domes were added per mould and sealed with 200 µl HistoGel. Samples were placed on ice for 10 minutes for the HistoGel to solidify and the resulting HistoGel blocks were then placed into histology cassettes and fixed in neutral buffered 10% formalin (Sigma-Aldrich) before being processed as described above for animal tissue sections. Histology sections were imaged using a Zeiss Axio Observer 7 microscope using an Axiocam 305 colour camera and ZenBlue (version 3.3) as an operating system.

### *Ostertagia ostertagi* Third Stage Larvae (L3) Exsheathing and Labelling

*Ostertagia ostertagi* L3 (Moredun isolate MOo2) were exsheathed and labelled following a previously published protocol used for *T. circumcinta* exsheathment and lipophilic dye labelling (Smith et al., 2021), as follows: a 15 ml Falcon tube containing 9 ml of Earle’s balanced salts solution (EBSS) was preheated in a water bath to 37°C and CO_2_-saturated over 1 h using an incubator tube connected to a CO_2_ tank. Approximately 1 x 10^5^ *O. ostertagi* L3 in 1 ml of tap water were added to the CO_2_-saturated EBSS and the sample continued to be saturated for a further 15 minutes. The Falcon tube was then sealed with Parafilm M and inverted six times before being placed horizontally into an incubator at 37°C, 5% CO_2_ for 4 h. The whole sample was then transferred into a 25 cm^2^ vented cap flask and incubated overnight at 37°C/5% CO_2_. The following day, the exsheathment of L3 larvae was monitored by light microscopy. The larvae were then washed four times by repeated centrifugation at 330 x *g* for 2 minutes and re-suspension in 50 ml of distilled water (pre-warmed to 37°C). After the final wash, the exL3 larvae were resuspended in 1 ml of sterile water and transferred to a microcentrifuge tube. ExL3 were fluorescently labelled by the addition of 2 µl PKH26 lipid dye (1 mM stock concentration) from the MINI26 PKH26 Red Fluorescent Cell Linker Kit (Sigma-Aldrich) and mixed by pipetting. Parasites were incubated with the dye for 15 minutes at room temperature, protected from light. Excess dye was removed by washing the larvae five times with distilled water, as described above, before finally re-suspending them in 1 ml of complete IntestiCult Growth Medium.

### *Ostertagia ostertagi* L3-Organoid Co-Culture

Abomasum organoids were cultivated in Matrigel™ for 7 days in an 8-well Nunc Lab-Tek II Chamber Slide System (Fisher Scientific), as described above. IntestiCult Growth Medium was removed and replaced with 250 µl of fresh pre-warmed complete IntestiCult Growth Medium. Fifty microliters of IntestiCult Growth Medium containing ~50 PKH26 labelled *O. ostertagi* were added to each well of organoids and kept at 37°C, 5% CO_2_ for 24-48h. After observing successful invasion into organoids, samples were fixed and prepared for immunofluorescent labelling as described above, with the difference of F-acting labelling by Phalloidin-iFluor 488 (ab176756, Abcam, 1:1000). Images of exL3 worms within and exiting organoids were captured using a Zeiss LSM880 Inverted Confocal Microscope and Zeiss Zen Black operating software. Light microscopy of *O. ostertagi* within organoids was performed to assess the length of time worms colonised the organoid lumen. After adding exL3 into wells containing organoids, samples were checked hourly to capture the earliest invasion and selected organoids were imaged overnight at a frequency of 10 minutes/image. Time-lapse microscopy was performed in an environmental control chamber at 37°C, 5% CO_2_ on a Zeiss Axiovert 200M microscope using Axiovision as operating software.

### *Ostertagia ostertagi* Comparative Invasion Assay

Bovine abomasal organoids were grown (as described above) until day 7 post-seeding. IntestiCult Growth Medium media was removed and 1 ml of ice-cold advDMEM/F12 was added to each well to liquify the Matrigel™. Resuspended organoids were pooled from 2 wells and transferred into a 15 ml Falcon tube on ice for 5 minutes for the organoids to settle. The supernatant was removed and organoids were carefully resuspended into 100 µl advDMEM/F12 to avoid breakage. One hundred microliters of resuspended organoids were added to 150 µl Matrigel™. For the negative control, Matrigel™ domes, one-hundred microliters of advDMEM/F12 were mixed with 150 µl Matrigel™ and used in parasite comparative invasion assays. Nunc Cell-Culture Treated 12-well plates (ThermoFisher) were seeded with organoid-positive and organoid-negative 40 µl Matrigel™ domes positioned at opposite sides of the well and incubated at 37°C, 5% CO_2_ for 10 minutes for the gel to polymerise. Afterward, 2 ml of IntestiCult Growth Medium media were added, followed by 50 µl IntestiCult Growth Medium containing ~200 exL3 worms and incubated at 37°C, 5% CO_2_ for 72h. To assess specific invasion into either control or organoid-containing Matrigel™ domes, all media were removed and transferred into a fresh 6-well collection plate. Each well was rinsed with 1 ml of PBS to remove larvae sticking to Matrigel™ domes and transferred into their respective collection well and 10 µl of 0.05 mol/l Iodine solution (Merck, Germany) was added to immobilize nematodes for counting non-invading individuals. The number of exL3 worms present within Matrigel™ domes and organoids was also enumerated. Successfully invading exL3 were normalized as a percentage of total larvae in each well and successful invasion into an organoid was normalised to total exL3 within the respective Matrigel™ dome. The comparative invasion assay was performed twice, with each plate each containing 8 replicate wells which were counted independently. As data were not normally distributed, significant differences between control or organoid-containing Matrigel™ domes were determined using non-parametric Mann-Whitney U Test using GraphPad Prism software (v8.0).

### *Ostertagia ostertagi* Larval Impact on Organoid Phenotype

*Ostertagia ostertagi* L3 larvae were exsheathed as described above and were subsequently incubated in 1 ml IntestiCult Growth Medium for 24 h at 37°C, 5% CO_2_. Bovine abomasal organoids were grown in Nunc Lab-Tek II Chamber Slides (Fisher Scientific) for 7 days using 400ul of IntestiCult Growth Medium. On the day of the experiment, the media was replaced with 300 µl of IntestiCult Growth Medium. The exL3 overnight cultures were diluted to ~1 exL3/µl and 100 µl added to a well containing organoids, with 100 µl exL3-free IntestiCult Growth Medium added to the control wells, to a final volume of 400 µl. Entire wells were imaged using a 36 tile overview scan and all wells were imaged once per hour for 23 h using the EC Plan-Neofluar 5x/0.16 Ph1 M27 objective on a Zeiss AxioObserver 7 microscope and the ZenBlue (v3.3) operating software. After the end of the experiment, overview tile images were stitched together using an automated ZenBlue stitching process. A total of 25 representative organoids were randomly selected from each well and processed into separate time-lapse stacks to be exported as.tiff files for analysis. Individual organoid stacks were analysed by measuring the total organoid surface area for each time point using the Organoseg standalone software (Borten et al., 2018). Total surface in pixel was normalised relative to t0 = 100%, to determine surface area changes over the time course of the experiment. The distribution of data was tested for normality variance using D’Agostino & Pearson test and significant differences were determined using Two-way RM ANOVA in GraphPad Prism software (v8.0).

### *Ostertagia ostertagi* Excretory/Secretory Product Impact on Organoid Phenotype

*Ostertagia ostertagi* L3 larvae were exsheathed as described above and then incubated for an additional 24 h in 5 ml IntestiCult Growth Medium overnight at 37°C, 5% CO_2_. The following day 4 ml of the exL3 culture supernatant were collected (“conditioned media”) and residual exL3 worms pelleted at 8000 x *g* for 3 min. The supernatant was collected and residual exL3 removed by passing through a Minisart NML sterile syringe filter, pore size 5.0 µm (Sartorius). Half of this “conditioned medium” containing parasite excretory and secretory (ESP) products was heat-inactivated (Hi-ESP) at 90°C for 40 minutes. For each experiment, culture media was removed from bovine abomasal organoids that had been growing for 14 days in Nunc Lab-Tek II Chamber Slides (Fisher Scientific) and replaced with 300 µl of fresh IntestiCult Growth Medium. One hundred exL3 worms in 100 µl of IntestiCult Growth Medium were added to an organoid-containing well. One-hundred microliters of conditioned media (containing parasite ESP) were added to a separate well containing organoids. A third well received 100 µl of Hi-ES and a fourth organoid well received 100 µl IntestiCult Growth Medium only (negative control). The four wells were imaged every hour and the entire organoid containing Matrigel™ domes were imaged using a 30 tile overview scan of the wells. Images were collected over 10 hours using the EC Plan-Neofluar 5x/0.16 Ph1 M27 objective on a Zeiss AxioObserver 7 microscope, using the operating software ZenBlue (v3.3). Surface area analysis was performed as described above on 25 randomly selected individual organoids for control, ESP, Hi-ESP, and exL3 treated organoids. Total surface in pixel was normalised relative to t0 = 100%, to determine surface area changes over the time course of the experiment. The distribution of data was tested for normality variance using D’Agostino & Pearson test and significant differences were determined using Two-way RM ANOVA followed by multiple comparisons tests using Šidák correction in GraphPad Prism software (v8.0).

## Results

### Establishment of Bovine Abomasum Organoids

**Figure 1:**
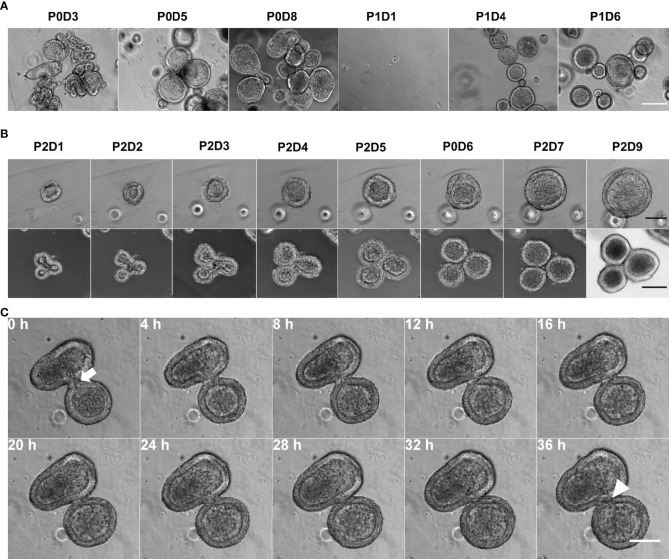
Establishment of *in vitro* bovine abomasum organoids. **(A)** Phase contrast images of bovine abomasum organoids during passage 0 and 1 (P0 and P1). Representative images were taken on indicated day DX of each passage. Scale bar = 200µm. **(B)** Growth of and development of representative passage 2 (P2) abomasal organoids from day 1 (D1) to day 9 (D9) following passage. Top panel shows phase contrast images and bottom panel shows differential interference contrast images of representative organoids. Scale bar = 100 µm. **(C)** Phase contrast time lapse of a budding organoid over 36h, with the shared luminal space (white arrow) closing off (white arrowhead). Scale bar = 100 µm.

Epithelial cells were extracted from the gastric fundic mucosa of three male 4 months old calves (one Aberdeen Angus and two Holstein-Friesians) were successfully embedded in Matrigel™. After seeding the crypts into culture (passage 0; P0), organoids of varying sizes and branched shapes formed, with the majority of organoids retaining a spherical structure from passage 1 (P1) onwards (**Figure 1A**). After passaging, smaller organoid fragments developed into spherical organoids containing a central lumen (**Figure 1B**, top row). Larger crypt fragments formed branched structures with a shared lumen but started budding into smaller spherical organoids (**Figure 1B**, bottom row). Complete budding occured between days 5 and 8, with separate distinct spherical organoids being formed (**Figure 1C**; **Supplementary video 1**). Abomasal organoids were cryopreserved at days 7-9, resuscitated, and able to resume organoid formation upon subsequent culture and passaging (**Supplementary Figure 1**). Organoids could be cultured up to at least passage 7 without losing their potential for self-organisation. Furthermore, cryopreserved organoids were successfully resuscitated after 12-months storage at -196°C, confirming their long-term storage potential.

### Epithelial Cell Markers and Gastric Morphology of Abomasal Organoids

**Figure 2:**
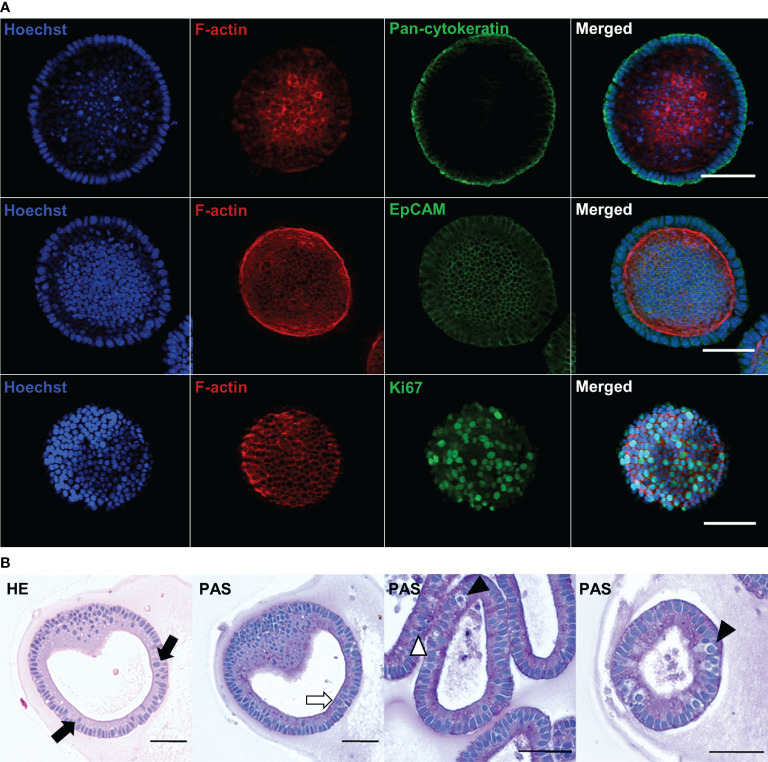
Immunofluorescence and histological characterisation of passage 2 (P2) bovine abomasum at day 7 to day 9 (D7 – D9) of *in vitro* culture. **(A)** Representative immunofluorescence images of abomasum organoids stained with either the epithelial cell markers pan-cytokeratin (green) and EpCAM (green) or the cell proliferation marker Ki67 (green), in addition to F-actin (red) and Hoechst (blue). Final panel shows merge of green, red and blue channels. Images for each antibody staining were taken at a different focal plane. Scale bars = 50 µm. **(B)** Histological images of organoid sections stained with haematoxylin or eosin (HE) and Periodic Acid–Schiff (PAS). Organoid sections show the spherical structure of the bovine abomasum organoids with a central lumen. Example polarised columnar epithelial cells with the apical surface facing the organoid lumen are highlighted (black arrows) indicating the presence of the glycocalyx and/or mucus layer (white arrow). Some cells show epithelial cell layer, having larger nuclei and brighter cytoplasm (black arrowheads) or over smaller cell sizes (white arrowhead) indicating the presence of different cell types. Scale bars = 50 µm.

IHC analysis was performed to investigate the microarchitecture and the presence of specific cell markers in the bovine abomasum organoids. The organoid epithelia comprised a ring of EpCAM+ and pan-cytokeratin+ positive cells implying the differentiation of the intestinal stem cells into epithelial cell types (**Figure 2A**, upper and middle row). F-actin labelling confirmed the orientation of the apical surface towards the interior lumen of the organoids (**Figure 2A**, middle row). F-actin staining of budding organoids confirmed the light microscopy observation of a shared luminal connection of day 7 organoids (**Supplementary Figure 3**). Immunostaining also revealed the presence of abundant Ki67-positive cells indicative of cell proliferation (**Figure 2A**, lower row). Histological sectioning and staining with HE and PAS further confirmed the self-organisation into an epithelial cell layer, as well as the turnover of cells in the epithelium by continuous shedding into the organoid lumen (**Figure 2B**, black arrow). PAS staining indicated the presence of polysaccharides on the apical surface within the organoids (white arrow) consistent with the presence of a glycocalyx and/or a thin mucus layer, in addition to different cell morphologies, suggesting the presence of multiple cell types within the organoids (arrowheads).

### Transcriptional Analysis of Bovine Abomasum Tissue and Organoids

**Figure 3:**
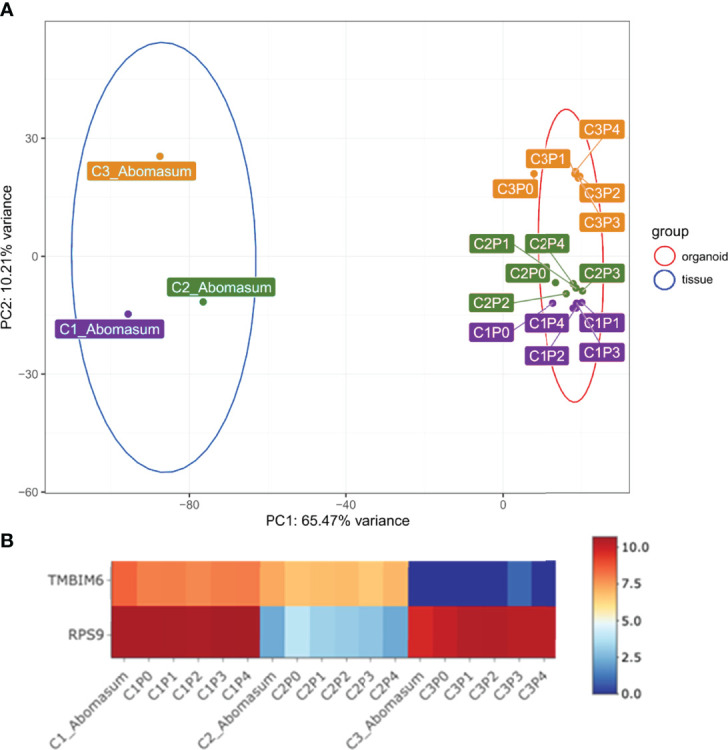
**(A)** Principal component analysis (PCA) of RNA-seq expression of the top 500 most variant genes in bovine abomasum tissue and abomasum organoids from three animals. Abomasum tissue and organoids we derived from Calf 1 (Aberdeen Angus; C1, purple), Calf 2 (Holstein-Friesian; C2, green), Calf 3 (Holstein-Friesian; C3, orange). Sample type, either tissue or organoid, and organoid passage number (passage 0 – 4; P0 – P4) are indicated in the figure. Ellipses indicate 95% confidence intervals for each cluster. **(B)** Heat map showing expression of representative genes (*TMBIM6* and *RPS9*) in abomasum tissue and derived abomasum organoids over serial passage. Colours indicate level of expression from high (red) to low (blue). The read count data for **(A)** and **(B)** was normalised using the median of ratios method from the DESeq2 package.

We next use mRNA-seq analysis to compare the gene expression profiles of abomasum tissue with those of organoids (P0 – P4) prepared from three individual cattle. Initially, expression profiles of the top 500 most variably expressed genes among these 18 individual samples were compared by principal component analysis (PCA) (**Figure 3A**). PCA analysis based on the differential gene expression clustered the samples into tissue and organoid groups, respectively. PCA analysis also confirmed that the gene expression profiles were consistent in organoids across multiple passage numbers, with little variation between samples (**Figure 3A**). While principal component 1 (accounting for 65.47% variance) showed samples clustering by sample type (tissue vs. organoid), samples were also organized on principal component 2 (accounting for 10.21% of variance) by sample source (individual cattle). This implied that the abomasum organoids maintained a gene expression profile associated with the individual animal from which they are derived (**Figure 3A**). Intra-animal variation was further interrogated to investigate animal specific gene expression in established organoids (**Supplementary figure 4**). While the majority of gene expression patterns were similar between the animal tissues and organoids, *TMBIM6* and *RPS9* showed animal specific variation of expression (**Figure 3B**). Excluding these two genes from the PCA analysis resulted in a breed-specific clustering of the two Holstein-Friesian calves (C2, C3) vs. the Aberdeen Angus calf (C1) (**Supplementary Figure 10**). This revealed that abomasum organoids retained specific gene expression profiles associated with the individual animal from which they are derived and that this was retained across multiple organoid passages.

### Expression of Cell- and Tissue-Specific Genes in Bovine Abomasum Organoids and Tissue

**Figure 4:**
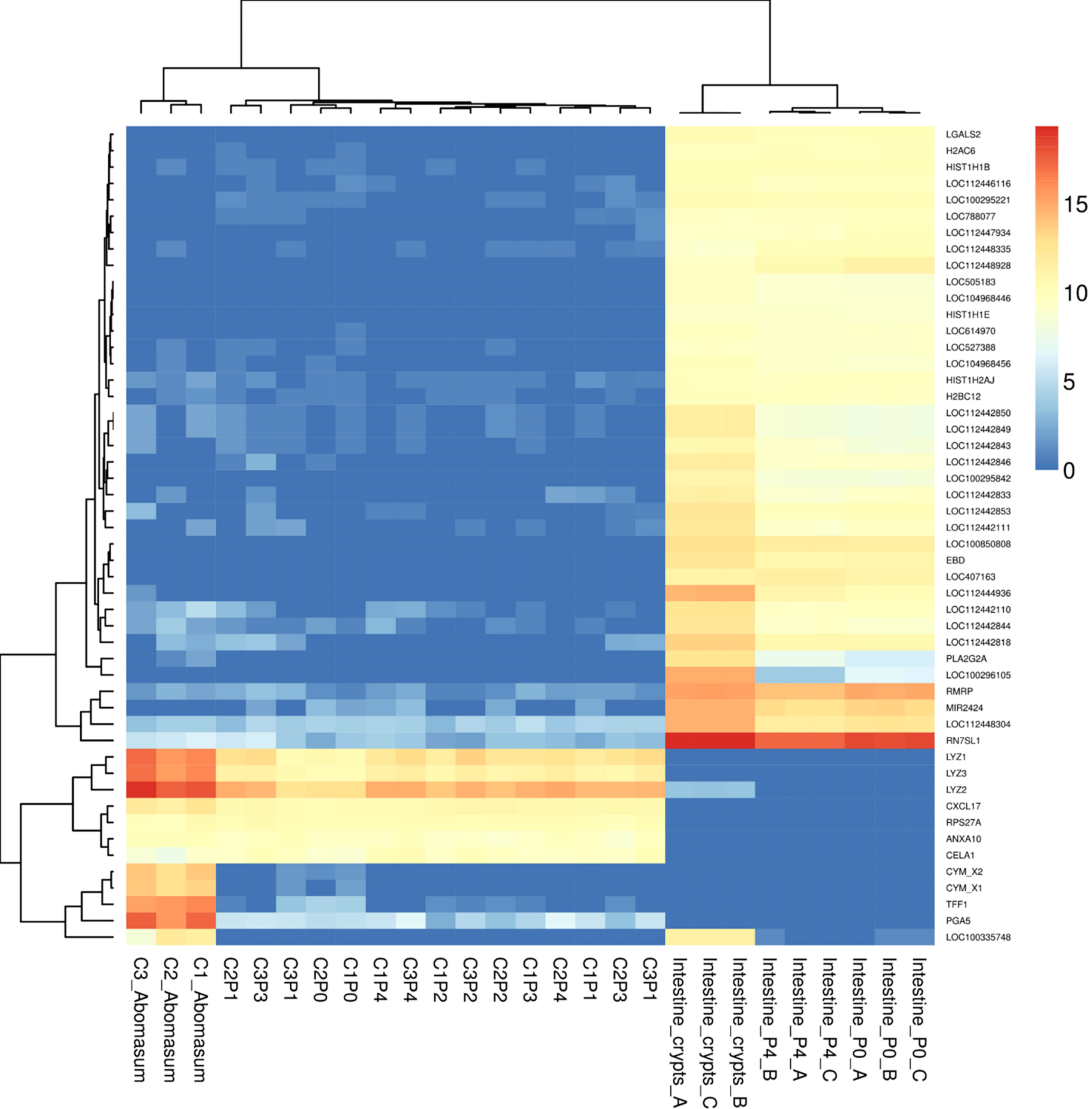
Heat map showing expression level of top 50 most variant genes from bovine abomasum and ileum tissue, abomasum organoids. Abomasum tissue and organoids we derived from Calf 1 (Aberdeen Angus; C1) and Calf 2,3 (Holstein-Friesian; C2, C3). Organoids are from serial passage number (passage 0 – 4; P0 – P4) as indicated in the figure. Colours indicate level of expression from high (red) to low (blue). The read count data was normalised using the median of ratios method from the DESeq2 package. The dendrograms indicate similarity between samples and gene expression profiles. Details of genes included in the heat map, are shown in **Supplemental File 1**.

**Figure 5:**
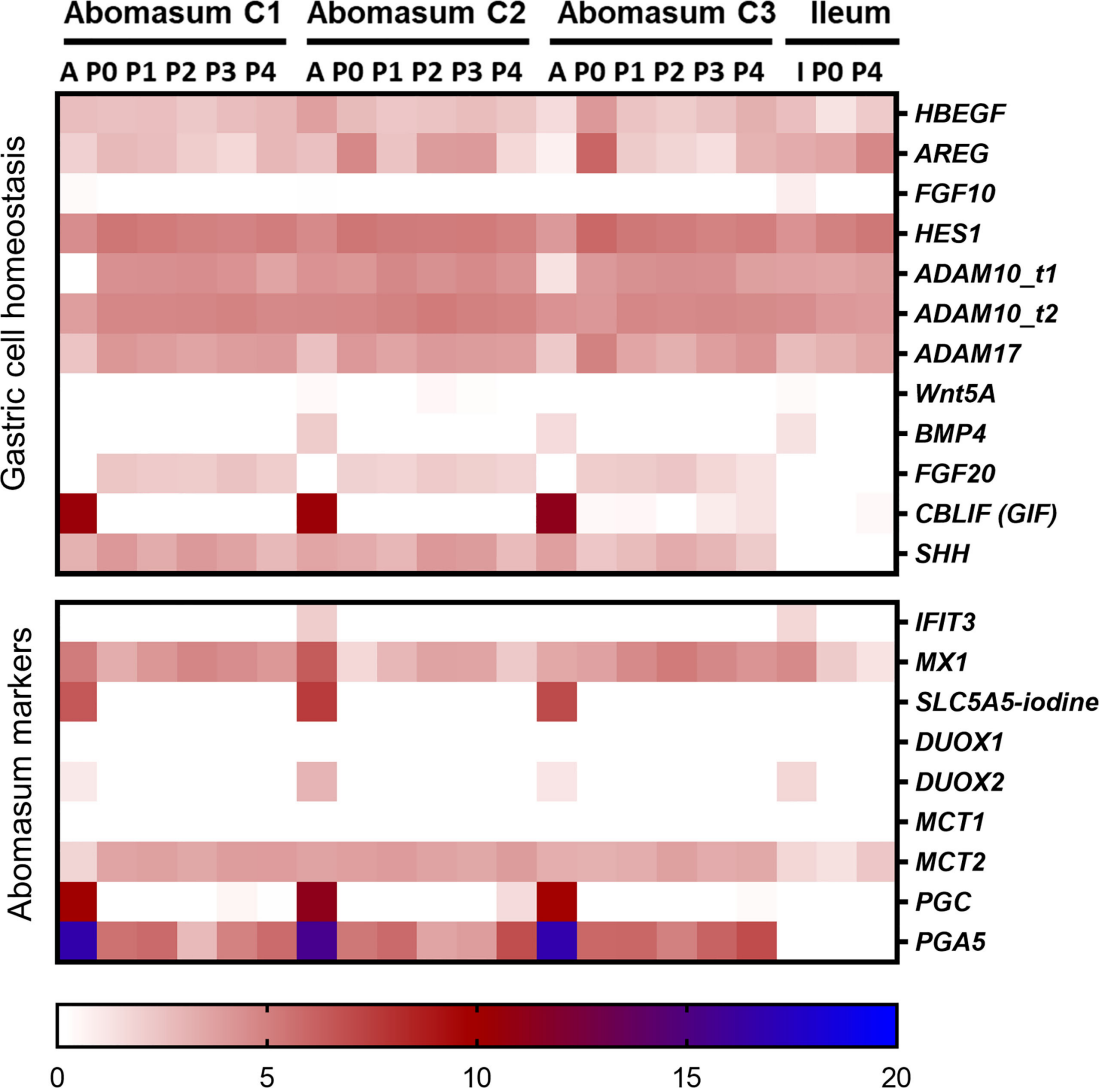
Heat map showing the expression of genes associated with gastrointestinal epithelia. RNA-seq analysis was performed to compare gene expression in abomasal and intestinal tissue and respective organoids across multiple passages. For abomasum samples, expression data is grouped by animal (calf 1 – 3, C1 – C3) and includes abomasum tissue (A) and abomasum organoids over serial passage (passage 0 – 4; P0 – P4). For ileum samples, expression data is from ileum tissue (I) and organoids from passage 0 (P0) and passage 4 (P4). The data was normalised by log_2_ transformation of transcripts per million reads. Details of genes included in the heat map are shown in **Supplemental File 1**.

We next sought to determine whether the organoids shared the expression of genes specifically associated with abomasum tissue. For this analysis, we compared gene expression data from the 18 individual abomasum organoid and abomasal tissue samples to previously published RNA-seq data from bovine intestinal (ileum) crypt and ileum organoids (GSE112674) (Hamilton et al., 2018). Analysis of the top 50 most variably expressed genes across these sample types revealed 45 genes specifically expressed in either ileum organoids, abomasum organoids, or abomasal tissue (**Figure 4**). This analysis identifies a panel of genes enriched for bovine abomasum (gastric stomach), including *ANXA10*, *CELA1*, *CXCL17*, *CYM_X1*, *CYM_X2, LYZ1-3*, *PGA5, RPS27A*, and *TFF1* (**Figure 4**; **Supplementary Figure 6**). Several genes associated with gastric cell homeostasis were expressed in abomasum tissue and abomasal organoids at similar levels, including *HBEGF*, *AREG*, *HES1*, *ADAM10*, *ADAM17*, and *SHH* (**Figure 5**). *SHH* was enriched abomasum organoids and tissue but absent in bovine ileal crypts or organoid data sets (**Figure 5**). *CBLIF* was expressed in abomasum tissue in all three cattle but was downregulated in abomasum organoids. Conversely, *FGF20* was expressed in abomasum organoids only and was not expressed in abomasum tissue from all three cattle (**Figure 5**). The genes *SLC5A5*, *DUOX2*, *PGC*, and *PGA5* were all specifically expressed in abomasum tissue. All four genes are expressed in abomasum tissue and not in the ileal crypt dataset, except for *DUOX2* which is also reportedly expressed in ileal crypts. Of these four genes, *PGA5* was also expressed in abomasum organoids (and absent in the ileal organoid dataset) (**Figure 5**). *MX1* and *MCT2* were expressed in abomasum tissue and abomasum organoids at similar levels. Both markers show a lower level of expression in ileal organoids (**Figure 5**).

**Figure 6:**
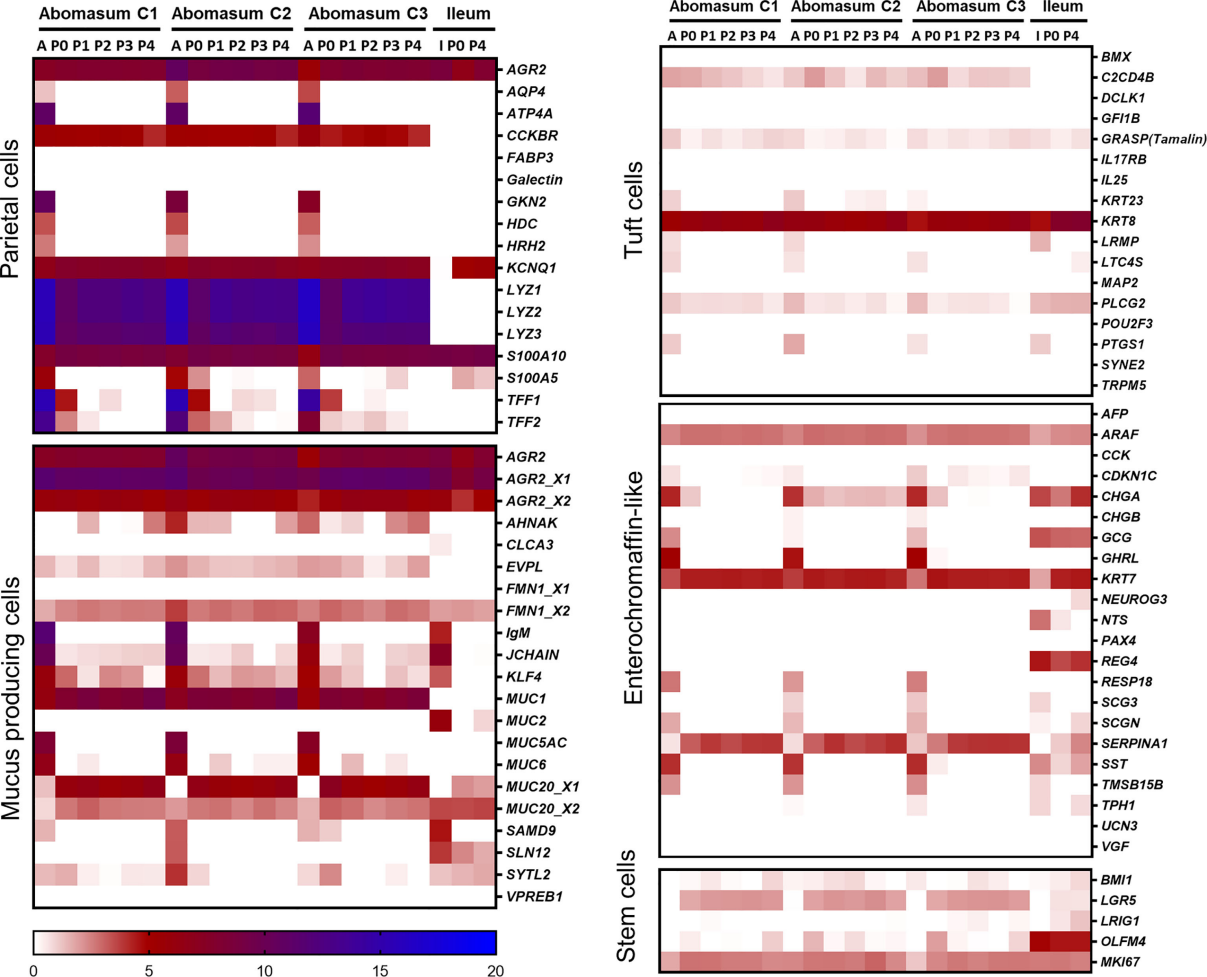
Heat map showing the expression of gastric cell specific markers from abomasum, ileum tissue and derived organoids. RNA-seq analysis was performed to compare gene expression in abomasal and intestinal tissue and respective organoids across multiple passages. For abomasum samples, expression data is grouped by animal (calf 1 – 3, C1 – C3) and includes abomasum tissue (A) and abomasum organoids over serial passage (passage 0 – 4; P0 – P4). For ileum samples, expression data is from ileum tissue (I) and organoids from passage 0 (P0) and passage 4 (P4). The data was normalised by log_2_ transformation of transcripts per million reads. Details of genes included in the heat map are shown in **Supplemental File 1**.

Gastric cell type-specific markers associated with either parietal cells, mucus producing cells, tuft cells, enterochromaffin-like cells, or stem cells were analysed for their expression in abomasum tissue and organoids (**Figure 6**). The lysosome genes *LYZ1*, *LYZ2*, and *LYZ3*, as well as the *CCKBR* gene, were found to be major parietal cell markers highly expressed in both abomasum tissue and organoids and are absent in ileal the bovine ileal transcriptomic datasets. Other parietal cell markers including *KCNQ1*, *S100A10*, and *AGR2* were also expressed in abomasum tissue and organoids. However, it should be noted that *AQP4*, *ATP4A*, *GKN2*, *HDC*, *HRH2*, and *S100A5* were expressed in abomasum tissue and not organoids and *TFF1* and *TFF2* expression was lost in abomasum organoids in later passages (**Figure 6**). The major abomasal-specific mucus producing cell marker, *MUC1*, was expressed in both abomasum tissue and organoids and as anticipated, this was absent in the ileal transcriptomic datasets. *AHNAK* and *EVFL* were also expressed at low levels, specifically in abomasum tissue and organoid samples. *MUC5* was expressed in abomasum tissue but not abomasum organoids and *MUC6* was expressed at a lower level and was more variable in abomasum organoids (although completely absent in the bovine ileal datasets) (**Figure 6**). The tuft cell specific marker *POU2F3* was not detected in any sample. However, *C2CD4B* is an ovine tuft cell-associated gene that was found to be expressed in abomasum tissue and organoids but was absent in the ileal datasets. *KRT8* was found to be highly expressed in abomasum tissue and organoids. In ileal crypts and organoids, GRASP and PLCG2 were also expressed in each of these sample types, albeit at a lower level in organoids than in tissue (**Figure 6**). The enterochromaffin-like cell markers *ARAF*, *CHGA*, *KRT7*, and *SERPINA1* were expressed in abomasum tissue and organoids. *NTS* and *REG4* are enterochromaffin cell-like markers present in the ileal datasets but not in the abomasum tissue and organoid transcriptomes (**Figure 6**), indicating tissue-type enrichment associated with particular cell types present in gastrointestinal organoids. The stem cell markers *BMI1*, *LGR5*, *OLFM4*, and *MKI67* were all detectable in abomasum organoids, although only *BMI1* and *MKI67* were detected in abomasum tissue samples (**Figure 6**). *OLFM4* was expressed at a higher level in the intestinal datasets compared to abomasum tissue or organoids, again indicating tissue-enrichment among these cell type markers.

The expression of numerous cell junction markers was conserved between abomasum tissue and abomasal organoids (across multiple passages), including genes associated with gap junctions, adherens junctions, tight junctions, and desmosomes (**Supplementary Figure 7**). We identified some cell junction encoding genes with expression patterns that suggested they were tissue specific. For example, the desmosome-related gene *JUP* and the tight junction-associated gene *CLDN15* were expressed in ileal crypts and organoids but not in the abomasum tissue or organoid data sets (**Supplementary Figure 7**). The expression profiles of immune-related genes, including toll-like receptors (TLRs), c-type lectin receptors (CLRs), chemokines, and cytokines were similar between the abomasum tissue and abomasal organoids (**Supplementary Figure 8**). Examples include the CLR gene *MCL-1*, the chemokines *CXCL16* and *CCL20* (the latter of which was found to be more highly expressed in all abomasum samples analysed here, compared to ileal samples), and the cytokine genes *CISH*, *IFNGR1*, *TGFBI*, *LTB_X1*, and *TNFRSF1B.* The cytokine genes *IL27A* and *IL4I1* were expressed in tissue collected from the Aberdeen Angus (C1) and all organoid samples (**Supplementary Figure 8**).

### Organoids as an *In Vitro* Infection Model for GI-Nematodes

**Figure 7:**
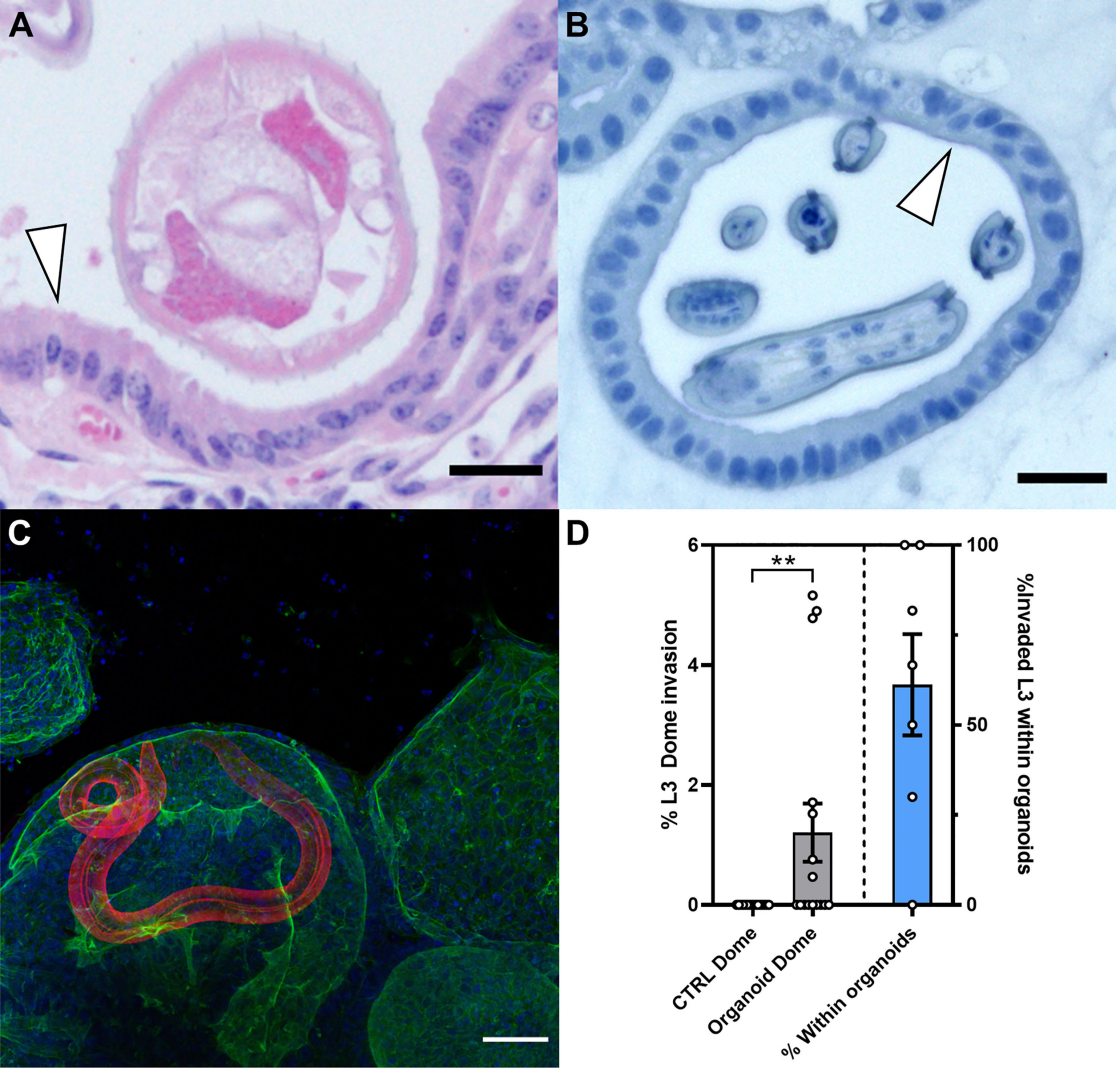
*Ostertagia ostertagi* invasion of bovine abomasal epithelial tissue and abomasum organoids after 24 h. **(A)** Hematoxylin and eosin (HE) stained section of a bovine abomasal gland infected with the parasitic nematode *O. ostertagi* at 21 days post infection. White arrowhead indicates the apical side of the gland epithelium, scale bar = 25 µm. **(B)** HE stained section of an exsheathed *O. ostertagi ex*L3 inside the lumen of a passage 7 bovine abomasum organoid at 24 h post infection. White arrowhead indicates the apical side of the organoid epithelia, scale bar = 25 µm**. (C)** Z-stack of a bovine abomasum organoid infected with a single exsheathed *O. ostertagi ex*L3 worm. Fluorescent labelling: *O. ostertagi ex*L3 (red), F-actin (green) and Hoechst (blue). Scale bar = 50 µm. **(D)** Quantification of comparative invasion of exsheathed *O. ostertagi* L3 into naïve Matrigel™ domes and Matrigel™ domes containing bovine organoids after 72 h. Bar graph (grey) shows percentage of total worms successfully invaded into Matrigel™ dome without organoids (CTRL Dome) and Matrigel™ dome containing bovine abomasum organoids (Organoid Dome). Results are normalised to 100% of total worms added to each well, n=16, **p<0.01, *Mann Whitney test).* Bar graph (blue) shows the percentage of worms that enter the Matrigel™ dome that are found within the lumen of the bovine abomasum organoid.

During infection with *O. ostertagi in vivo*, the gastric gland epithelium and parasitic nematode are in proximity (**Figure 7A**). We were able to mimic this close host-parasite relationship *in vitro* using bovine abomasum organoids infected with *O. ostertagi* exL3 stage larvae (**Figure 7B**). After *in vitro* exsheathing, approximately 100 exL3s were added into 8 well chamber slides containing organoids embedded in Matrigel™ with IntestiCult Growth Medium. ExL3s migrated into the Matrigel™ domes and subsequently invaded the organoids over the course of 24 h, with the earliest time point of organoid invasion documented after 4 h. Histological sections of the invaded organoids revealed a similar proximity of exL3 to the abomasal epithelium as we observed in adults within the abomasal glands physical representation of the *in vivo* host-parasite interface (**Figure 7B**). Parasites were able to invade the organoids from the basal epithelial surface and resided within the organoid lumen for several hours (**Figure 7C**; **Supplementary Video 2**). Representative Z-stack images through an infected organoid reveal the close contact of exL3 anterior proximal head regions with the apical surface of the organoid epithelia (**Supplementary Figure 7**). Within the organoids, some of the larvae displayed twitching circular movements within the lumen and appeared to be probing the epithelial surface with the anterior proximal end of the worm.

We next conducted a comparative invasion assay to determine whether the invasion of the larvae into the organoids was a specific response to factors or cues produced by the organoids or instead was due to their non-specific migration into the extracellular Matrigel™ matrix. In this assay, larval invasion of organoid-containing Matrigel™ domes was compared to domes without organoids in the same well. Empty and organoid-containing Matrigel™ domes were seeded in the same wells of a 6-well plate, with organoid and control domes approximately 5-6 mm apart and approximately 200 exL3 larvae added to each well. The wells were then analysed 72 h later. No larvae were present within the empty Matrigel™ domes that did not contain organoids. In contrast, 7 out of 16 of the Matrigel™ domes containing organoids also contained larvae (**Figure 7D**). Furthermore, of the exL3 invading a Matrigel™ dome, a mean of 61% ± 37% (SD) successfully invaded the lumen of organoids (**Figure 7D**).

**Figure 8:**
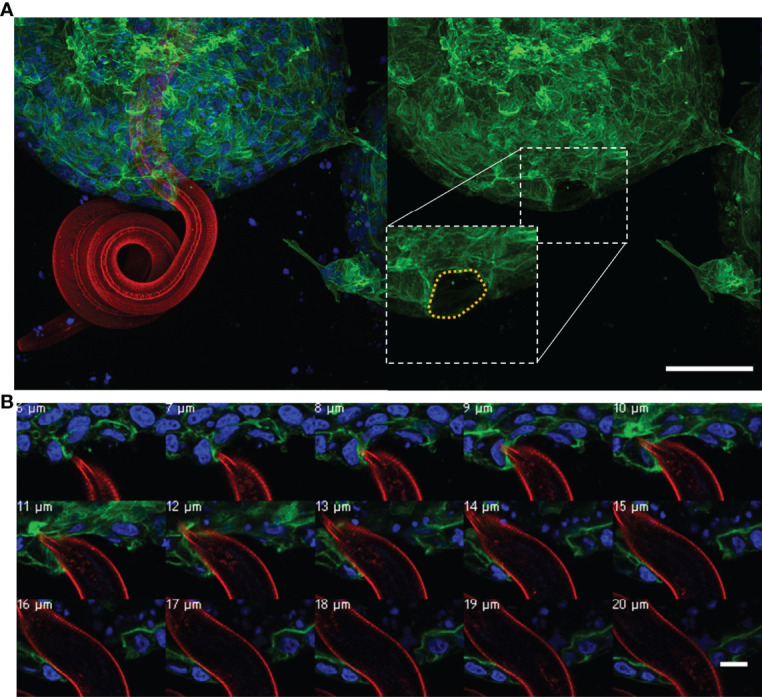
*Ostertagia ostertagi* exL3 exiting a bovine abomasal abomasum organoid. **(A)** Composite and individual channels of a Z-stack of *O. ostertagi* penetrating the apical surface of the organoid epithelium. **(B)** Magnified Z-stack series of the nematode pushing aside epithelial cells during exiting the organoid, Z-stack increments are listed represent depth within the Z-stack. Fluorescent labelling: *O. ostertagi ex*L3 (red), F-actin (green) and nuclear marker (blue). Panel **(A)**, scale bar = 50 µm. Panel **(B)**, scale bar = 10 µm.

We also observed some exL3 larvae exiting from the organoid lumen. The larvae were observed to probe the apical surface of the organoid epithelial layer and subsequently mechanically force their way between epithelial cells, creating a hole in the epithelial layer through which the entire exL3 larvae burrowed (**Figure 8A**). Higher magnification imaging of the organoid penetration by the larvae revealed the stretching of the organoid epithelium around the exL3 larval body (**Figure 8B**, **Supplementary Figure 11**).

### Abomasal Organoid Responses to *O. ostertagi* L3 and Their Products

**Figure 9:**
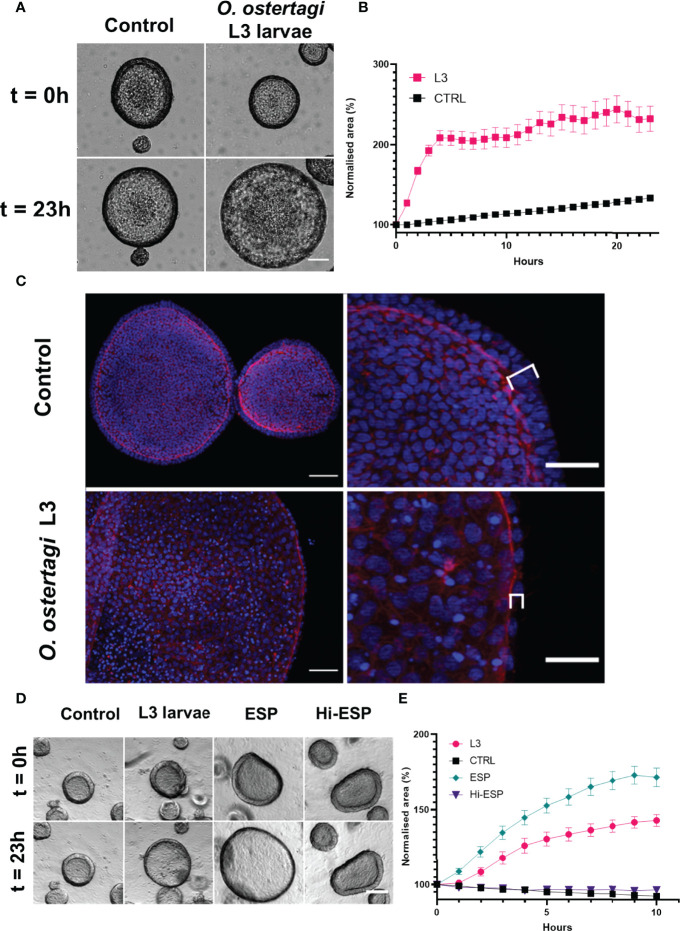
Characterisation of a ballooning phenotype in bovine abomasum organoids upon exposure to *Ostertagia ostertagi* exL3 larvae and associated excretory-secretory products (ESP). **(A)** Light microscopy analysis of day 7 bovine abomasal organoids exposed to *O. ostertagi* L3 larvae over 23 h. Representative examples of organoids at the indicated time points are shown. Scale bar = 100 µm. **(B)** Representative experiment of day 7 bovine abomasal organoid expansion upon treatment with *O. ostertagi* L3 larvae over time. Normalised organoid surface area is shown relative to t = 0 (pre-worm exposure) and at 1 h intervals over 23 h following worm exposure. Control organoids (CTRL) were grown in parallel and not exposed to *O. ostertagi* exL3. Surface areas are expressed as percentages relative to that at t = 0 (100%). Data shows one experiment with n = 25 randomly selected organoids for both control (CTRL) and worm exposed organoids, with each data point showing mean ± standard error of the mean (SEM). **(C)** Representative images of control (top row) and exL3 exposed (bottom row) organoids fluorescently labelled for F-actin (red) and Hoechst (blue). Enlarged images show the stretching and reduced width of organoid epithelial layer (brackets). Scale bar: Left column = 50 µm, right column = 25 µm. **(D)** Representative light microscopy images of day 14 bovine abomasal organoids exposed to *O. ostertagi* exL3 larvae, larval products (ESP) or heat inactivated products (Hi-ES). Representative examples at the indicated time points are shown. Scale bar = 100 µm. **(E)** Representative experiment of day 14 bovine abomasal organoid expansion upon treatment with *O. ostertagi* L3 larvae and ES products over time. Normalised organoid surface area is shown relative to t = 0 (pre-worm exposure) and at one-hour intervals over 10 h following worm exposure to *O. ostertagi* exL3 (L3), ESPs and Hi-ES. Control organoids (CTRL) were grown in parallel and not exposed to *O. ostertagi* exL3. Surface areas are expressed as percentages relative to that at t = 0 (100%). Data shows one experiment with n = 25 randomly selected organoids replicate organoids for CTRL, L3, ESP and Hi-ESP exposed organoids, with each data point representing mean ± SEM. Surface areas, expressed as percentages relative to that at t = 0 (100%).

When the abomasum organoids were exposed to *O. ostertagi* exL3 larvae a “ballooning” phenotype was observed in the organoids in which the organoid rapidly expanded in size. Live cell imaging of organoid cultures exposed to exL3 larvae over a 23-hour period showed the rapid onset of this ballooning phenotype. The organoids were found to increase in size within 30-60 minutes of exposure and that this increase would plateau by 4 h of co-culture (**Figures 9A, B**). Specifically, organoids exposed to exL3 larvae were found to be more than double in size over the 23 h study period (**Figure 9B**). By comparison, in control treated organoids that were not exposed to exL3 larvae, only a modest size increase was observed during the 23 h culture period (mean increase of 116%; **Figure 9B**). This was reflected in a significant effect of treatment (p<0.0001), time (p<0.0001), and treatment × time interaction (p<0.0001) by two-way ANOVA. At the end of the 23 h culture period, the organoids were fixed and counterstained to identify cell nuclei and F-actin to assess their morphology. The epithelium of the “ballooned” organoids was thinner than that of negative control treated organoids (**Figure 9C**). In addition, the cell nuclei were spaced further apart and lost their perpendicular orientation to the apical surface marked by the F-actin staining (**Figure 9C**). Together, this analysis suggests that exposure to *O. ostertagi* exL3 larvae induced changes in the organoids that caused their epithelial cells to become stretched.

To determine whether the ballooning phenotype was the result of an interaction between organoid epithelial cells and larval ES products, organoids were treated with sterile-filtered ESP generated from an overnight culture of exL3 larvae. Live imaging of organoids treated with ESP products showed a similar increase in organoid size that was observed after exposure of the organoids to live exL3 larvae (**Figures 9D, E**). Two-way ANOVA revealed that there was a statistically significant effect of treatment (p<0.0001), time (p<0.0001), and treatment × time interaction (p<0.0001). Multiple comparison analysis at 10 h post-treatment revealed that mean organoid size was significantly increased in live exL3 treated (p<0.0001) and ES product treated (p<0.0001) organoids compared to negative control organoids treated with media alone, with a mean increase of 142.8% and 171.4% for live exL3 and ESP treated organoids. Furthermore, the increase in organoid size was significantly greater in ESP treated organoids compared to exL3 exposed organoids (p= 0.0214). Heat inactivation of *O. ostertagi* exL3 ES prior to treatment resulted in no significant difference in organoid size compared to negative control organoids by 10 hr post-treatment (p= 0.1193) (**Figure 9E**). These data suggested that the ballooning caused in the organoids occurred in response to their exposure to functionally active, heat labile parasite-derived ES products.

## Discussion

The bovine abomasum is the site of infection for the economically important parasitic nematode *O. ostertagi*. However limited accessibility to this predilection site is a major hurdle to performing detailed studies of host-pathogen interactions *in vivo*. Here we describe the cultivation of the bovine abomasal organoids from abomasal gastric glands and demonstrate their feasibility as an *in vitro* GIN infection model. Furthermore, the bovine abomasal organoids reported here were cultivated using the same *in vitro* culture conditions and reagents as previously reported for other ruminant species and regions of the GI tract, demonstrating the ease with which published protocols can be adapted to different species and GI organoid types (Hamilton et al., 2018; Smith et al., 2021).

A major advantage of organoids over other tissue culture systems is their multicellular composition and tissue authenticity. This has been reported in previous characterizations of livestock GI organoids (Clevers, 2016; Hamilton et al., 2018; Beaumont et al., 2021; Kar et al., 2021; Nash et al., 2021; Smith et al., 2021) and was found to be the case for the bovine abomasum organoids reported here. Abomasum-derived gastric glands were found to self-organize into three-dimensional structures within which cells had differentiated into the different epithelial cell types typically found in the bovine abomasum. Moreover, the transcriptomic analysis revealed the expression of genes associated with gastric tissue in these organoids, demonstrating their tissue authenticity. A particular hurdle to determining the tissue authenticity of abomasal organoids is the lack of identified tissue markers for ruminant GI cell lineages. However, our approach here to compare transcriptomic profiles of abomasum tissue and organoids to previously published bovine ileal datasets help to identify markers enriched in particular regions of the GI tract. For example, our analysis showed three lysozyme genes (*LYZ1-3*), chymotrypsin-like elastase 1 (*CELA1*), cholecystokinin B receptor (*CCKBR*), pepsinogen A5 (*PGA5*), the chemokine ligand 17 (*CXCL17*), annexin A1 (*ANXA1*), and multiple mucins are all expressed in abomasal tissue samples and are absent in previously published bovine ileal transcriptomes (Hamilton et al., 2018). Importantly, we have shown that these genes are also expressed in organoids derived from bovine abomasal stem cells, demonstrating their distinct enrichment in this tissue. This is similar to our previous finding in ovine organoids in which these genes are expressed in abomasum tissue and organoids and not in ileum tissue or organoids (Smith et al., 2021). It has been reported that the gene expression of trefoil factors 1-3 (*TFF1-3*) and mucins 1, 6, and 20 (*MUC1*, *MUC6*, and *MUC20*) is upregulated in the bovine abomasum during *O. ostertagi* infection (Rinaldi et al., 2011). Expression of these genes peaks after adults emerge from gastric glands at 24 days post-infection, coinciding with mucosa hyperplasia and increased mucous production (Rinaldi et al., 2011). We found *TFF1-3*, *MUC1*, *MUC6*, and *MUC20* expression in the bovine abomasal organoids reported here, which is consistent with their feasibility as a relevant *in vitro* culture model for studying host responses to parasite infection. The expression of these particular mucin genes also suggests the presence of functional mucin secreting goblet cells that are associated with abomasum tissue. This is also supported by the presence of PAS-staining on the apical surface of organoid epithelial cells, although this staining could also represent the glycocalyx. It is therefore possible that the abomasal organoid model could be used to study the role and modulation of the mucus barrier during *O. ostertagi* infection *in vitro*, although further characterisation of mucin secretion would be required. Altogether, our results indicate the presence and differentiation (or partial differentiation) of each of these specific cell types in abomasum organoids. In addition to their gastric expression profiles, organoids retained a “donor-specific” expression profile, revealing individual and breed-specific heterogeneity. This transcriptomic heterogeneity is expected and reflects observations in human tissue derived organoids (Driehuis et al., 2020).

Several studies have harnessed the value of GI organoids to study host responses to GIN (Eichenberger et al., 2018b; Eichenberger et al., 2018a; Nusse et al., 2018; Luo et al., 2019; Duque-Correa et al., 2020; Hares et al., 2021; Cavallero et al., 2022; Drurey et al., 2022). However, these studies relied on the use of concentrated worm ES products to model host-parasite interactions, as opposed to live nematodes. While we recently reported the invasion of ruminant GI organoids by the sheep stomach worm *Teladorsagia circumcincta* (Smith et al., 2021), and the current study describes interactions between *O. ostertagi* and bovine gastric organoid tissue, our data show that the addition of *O. ostertagi* larvae to the organoid culture media is sufficient to elicit their invasion of the organoids, making organoid challenge with live nematodes very practical and efficient. The migration of the larvae into the Matrigel™ domes only occurred when the domes contained organoids, suggesting that the nematodes can sense the cues produced by the epithelial cells of the organoids and migrate towards them. Homing behaviour has previously been reported for the plant parasitic nematode *Meloidogyne incognita* (Hida et al., 2015) and the findings in the present study warrant further investigation, as underpinning the mechanism by which GIN nematodes sense the GI epithelia, or more specifically the glands and crypts, could identify new targets for disease intervention. Parasitic nematodes are able to sense the humidity, chemical, thermal, and mechanical makeup of their environment and are considered to use these factors when navigating host tissues (Gang and Hallem, 2016). While CO_2_ and temperature are important triggers for *Ostertagia ostertagi* L3 exsheathment, and the *in vitro* conditions used herein are considered to mimic those of the bovine rumen, the precise sensory mechanisms involved are not known. In our previous study, we observed multiple *T. circumcincta* nematodes within individual organoids, while other organoids remained uninvaded (Smith et al., 2021). In the current study *O. ostertagi* larvae displayed a similar invasive behaviour. This may suggest that the parasites can display a preference for certain organoids in which to invade.

In the present, study, *O. ostertagi* larvae exhibited a probing behaviour of the organoid epithelium at both the apical and basal surface. It is possible that this behaviour is displayed by GI nematodes *in vivo* to identify glands and crypts to invade. While GI nematodes interact with the apical surface of the GI epithelium *in vivo*, we observed basal and apical penetration into and out of our abomasal organoids by *O. ostertagi* and *T. circumcincta* larvae. We cannot discern whether this penetration behaviour reflects *in vivo* biology or is an artifact of our organoid model. While it is considered that gastric nematodes enter the glands through the gastric neck, it is possible that they also employ other strategies to enter and exit the glands, including migration into the sub-epithelial mucosa (Snider et al., 1988). Emergence from glands after moulting and developing into adults negatively impacts the integrity of the gastric mucosa adjacent to infected glands, causing cell junctions to separate (Murray et al., 1970). This is comparable to the exiting of an abomasal organoid documented here, in which an individual *O. ostertagi* worm was observed burrowing between epithelial cells, disrupting junctions to form a hole in the side of the organoid, suggesting a paracellular invasion in and out of organoids.

We observed a ballooning phenotype in which organoids became inflated in size and this could be induced by either exposure to either live exL3 worms or exL3 larvae ES products present in conditioned media. That treatment with a relatively low number of worms was sufficient to induce this phenotype without physical contact of the worms with the organoids and importantly shows that this response is not an artefact of highly concentrated parasite-derived products in the ESP preparation, but rather is likely to be a physiologically-relevant host tissue response to molecules released by the parasite. Moreover, heat inactivation of the ES products ablated this effect, suggesting that this ballooning phenotype was due to interactions between secreted heat-labile parasite product/s and receptor/s on epithelial cells in the organoids. Further study is necessary to determine the specific molecules involved and to underpin the mechanism of this interaction. It is plausible that the swelling observed in response to *O. ostertagi* exposure is due to an osmotic shift drawing fluid from outside of the organoids into the luminal space.

õ*In vivo*, nematode-infected gastric glands are similarly swollen, showing the stretching and flattening of epithelial cells (Murray et al., 1970; Snider et al., 1988) appearing similar to the cell morphologies associated with the organoid ballooning observed in response to exposure to *O. ostertagi* larvae. Our findings indicate this may be driven by the parasites themselves to create a suitable niche within which they can reside and develop. The ballooning phenotype here is similar to previous reports of murine intestinal organoids treated with ESP from the parasitic nematode *Heligmosomoides polygyrus*, in which the organoid luminal space was found to increase in response to ES treatment (Drurey et al., 2022). Intestinal organoids derived from *H. polygyrus*-infected mice also display hyper-proliferation and exhibit a large luminal space (Nusse et al., 2018). However, the increases in organoid size and luminal space observed after exposure to *H. polygyrus* ES products are distinctly different to those that occur after exposure to *O. ostertagi*, since the increase in organoid size following *H. polygyrus* ES treatment was associated with rapid epithelial cell proliferation rather than swelling.

Overall, we describe the development and characterisation of bovine abomasal organoids and demonstrate their utility as a physiologically relevant *in vitro* model for investigating the interactions of GIN with the abomasal epithelium. As a disruptive technology, these abomasal organoids provide a springboard for uncovering novel parasite behaviour and for interrogating precise mechanisms of host-parasite interactions in the bovine abomasal epithelium. We consider the abomasal organoid culture system described in this study provides a novel *in vitro* system than can be used to identify novel targets for drug and/or vaccine interventions aimed at controlling *O. ostertagi* and other important GIN infections in cattle.

## Data Availability statement

The datasets presented in this study can be found in online repositories. The names of the repository/repositories and accession number(s) can be found in the article/**Supplementary Material**.

## Ethics Statement

Ethical review and approval was not required for the animal study because all animal material used in this study was collected post-mortem from animals used in other studies, therefore no prior ethical approval was necessary.

## Author Contributions

MF, DS, DP, AN, LM, NM, and TM conceived the study. All authors designed the experiments. MF, DS, DP, and KH performed the work. MF, DS, and DP analysed the data. MF, DS, DP, AN, LM, NM, and TM wrote the manuscript. All authors read and approved the final manuscript.

## Funding

The work was supported by Zoetis as part of its partnership with the Easter Bush Research Consortium, with funding awarded to AN, LM, NM, and TM. Additional funding through Moredun Foundation research fellowships was awarded to DP and DS. PS, AN, and TM gratefully receive funding from the Scottish Government Rural and Environment Science and Analytical Services (RESAS). LM and NM were also supported by Institute Strategic Programme Grant funding from the BBSRC (https://bbsrc.ukri.org/; BS/E/D/20002173).

## Conflict of Interest

The authors declare that the research was conducted in the absence of any commercial or financial relationships that could be construed as a potential conflict of interest.

This study received funding from Zoetis. The funder had the following involvement with the study: the decision to submit the manuscript for publication.

## Publisher’s Note

All claims expressed in this article are solely those of the authors and do not necessarily represent those of their affiliated organizations, or those of the publisher, the editors and the reviewers. Any product that may be evaluated in this article, or claim that may be made by its manufacturer, is not guaranteed or endorsed by the publisher.

## Supplementary Material

Supplementary Figure 1 Light microscopy time-lapse imaging of a bovine abomasum gland forming an organoid over 17 h after passaging. Scale bar = 50 µm

Supplementary Figure 2 Negative controls for immunofluorescence antibody labelling in abomasum organoids (**Figures 2**). Representative microscopy images of abomasum organoids probed with non-specific host IgG followed by indirect Alexa Fluor^®^ 488-conjugated secondary antibody labelling (green), F-actin (red) and nuclear marker (blue). Labelling control for pan-cytokeratin (top, middle row) and Epcam (bottom row) organoid image represents. Scale bars = 50 µm.

Supplementary Figure 3 Multiple budding abomasal organoids sharing a luminal cavity. Composite and individual channels of a Z-stack orthogonal projection of a representative immunofluorescent image of abomasum oarganoids stained with the epithelial cell markers pan-cytokeratin (green), F-actin (red) and nuclear marker (blue). Scale bar = 100 µm.

Supplementary Figure 4 Heat map showing expression level of top 50 most variant genes from bovine abomasum and serial passaged abomasum organoids (P0-4). The read count data were normalised using the median of ratios method from the DESeq2 package. Colours indicate level of expression from low (blue) to high (red). The dendrograms indicate similarity between samples and gene expression profiles. Details of genes included in the heat map, are shown in **Supplemental File 1**.

Supplementary Figure 5 Principal component analysis (PCA) of the top 500 most variant genes comparing bovine abomasum and intestine tissue and organoids. The read count data were normalised using the median of ratios method from the DESeq2 package. Sample type is indicated in the key and includes: abomasum organoid (red); abomasum tissue (green); intestinal crypts (blue); intestine organoid (purple).

Supplementary Figure 6 Principal component analysis (PCA) of the top 50 most variant genes comparing bovine abomasum and intestine tissue and organoids. The read count data were normalised using the median of ratios method from the DESeq2 package. Sample type is indicated in the key and includes: abomasum organoid (blue); abomasum tissue (red); intestinal crypts (green); intestine organoid (purple).

Supplementary Figure 7 Heat map showing the expression of genes associated with cell junctions in abomasum and ileum tissue and organoids. RNA-seq analysis was performed to compare gene expression in abomasal and intestinal tissue respective organoids across multiple passages. Each square from left to right under “abomasum C1-3” and “Ileum” represent cows 1-3 and the pooled intestine samples, primary tissue (A,I) and passages P0-P4. The data was normalised by log_2_ transformation of transcripts per million reads. Details of genes included in the heat map are shown in **Supplemental File 1**.

Supplementary Figure 8 Heat map showing the expression of immune-related gene expression in abomasum and ileum tissue and organoids. RNA-seq analysis was performed to compare gene expression in abomasal and intestinal tissue respective organoids across multiple passages. Each square from left to right under “abomasum C1-3” and “Ileum” represent cows 1-3 and the pooled intestine samples, primary tissue (A,I) and passages P0-P4. The data was normalised by log_2_ transformation of transcripts per million reads. Details of genes included in the heat map are shown in **Supplemental File 1**.

Supplementary Figure 9 Individual Z-stack images of **Figures 7C**, showing an Ostertagia ostertagi exL3 inside an bovine abomasal organoid. Fluorescent labelling: O. ostertagi exL3 (red), F-actin (green) and nuclear marker (blue). Scale bar = 50 µm, images 1 µm apart.

Supplementary Figure 10 Principal component analysis (PCA) of RNA-seq expression of the top 500 most variant genes in bovine abomasum tissue and abomasum organoids from three animals, excluding TMBIM6 and RPSP9. Abomasum tissue and organoids are derived from Calf 1 (Aberdeen Angus; C1, purple), Calf 2 (Holstein-Friesian; C2, green), Calf 3 (Holstein-Friesian; C3, orange). The read count data were normalised using the median of ratios method from the DESeq2 package. Sample type, either tissue or organoid, and organoid passage number (passage 0 – 4; P0 – P4) are indicated in the figure. Ellipses indicates 95% confidence intervals for each cluster.

Supplementary Figure 11 3D representation of the entire Z-stack of Ostertagia ostertagi L3 penetrating the apical surface of the organoid epithelium (**Figures 8**). Stretched cells and nuclei surrounding the area of exit indicate a paracellular invasion (white arrowhead). Labelling: O. ostertagi exL3 (red), F-actin (green) and nuclear marker (blue).

Supplementary Video 1 Light microscopy time-lapse imaging of bovine abomasum organoids budding over a period of 40 hours. Scale bar = 100 µm

Supplementary Video 2 Light microscopy time-lapse imaging of an *Ostertagia ostertagi* exL3 larvae inside a bovine abomasal organoid over 530 minutes. Scale bar = 100µm.

Supplementary Video 3 Light microscopy time lapse video of day 7 bovine abomasal organoids with and without *O. ostertagi* exL3 larvae over 23h (**Figures 9A**). Scale bar = 100 µm.

Supplementary Video 4 Light microscopy time lapse video of naïve (CTRL) day 14 bovine abomasal organoids, exposed to *O. ostertagi* exL3 larvae, larval products (ESP) or heat-inactivated ES products (Hi-ES). Representative examples at the indicated time points are shown. Scale bar = 100 µm.

Supplementary File 1 Raw data used for RNA-seq analysis. (Sheet1) Total transcript per million (TPM) count data of pseudoalignments for bovine abomasal tissue, bovine abomasal organoids, bovine ileal crypts and bovine ileal organoids. (Sheet2) TPM data used for individual heat maps. (Sheet3) TPM of the top 50 differentially expressed genes between bovine abomasum tissue and bovine abomasal organoids. (Sheet4) TPM of the top 50 differentially expressed genes between bovine abomasum tissue, abomasal organoids, ileal crypts and ileal organoids.
