# Supplementary figures and images for: Development of Bovine Gastric Organoids as a Novel In Vitro Model to Study Host-Parasite Interactions in Gastrointestinal Nematode Infections
Source: Front Cell Infect Microbiol. 2022 Jun 30;12:904606. doi: 10.3389/fcimb.2022.904606 (PMC9281477; doi:10.3389/fcimb.2022.904606)

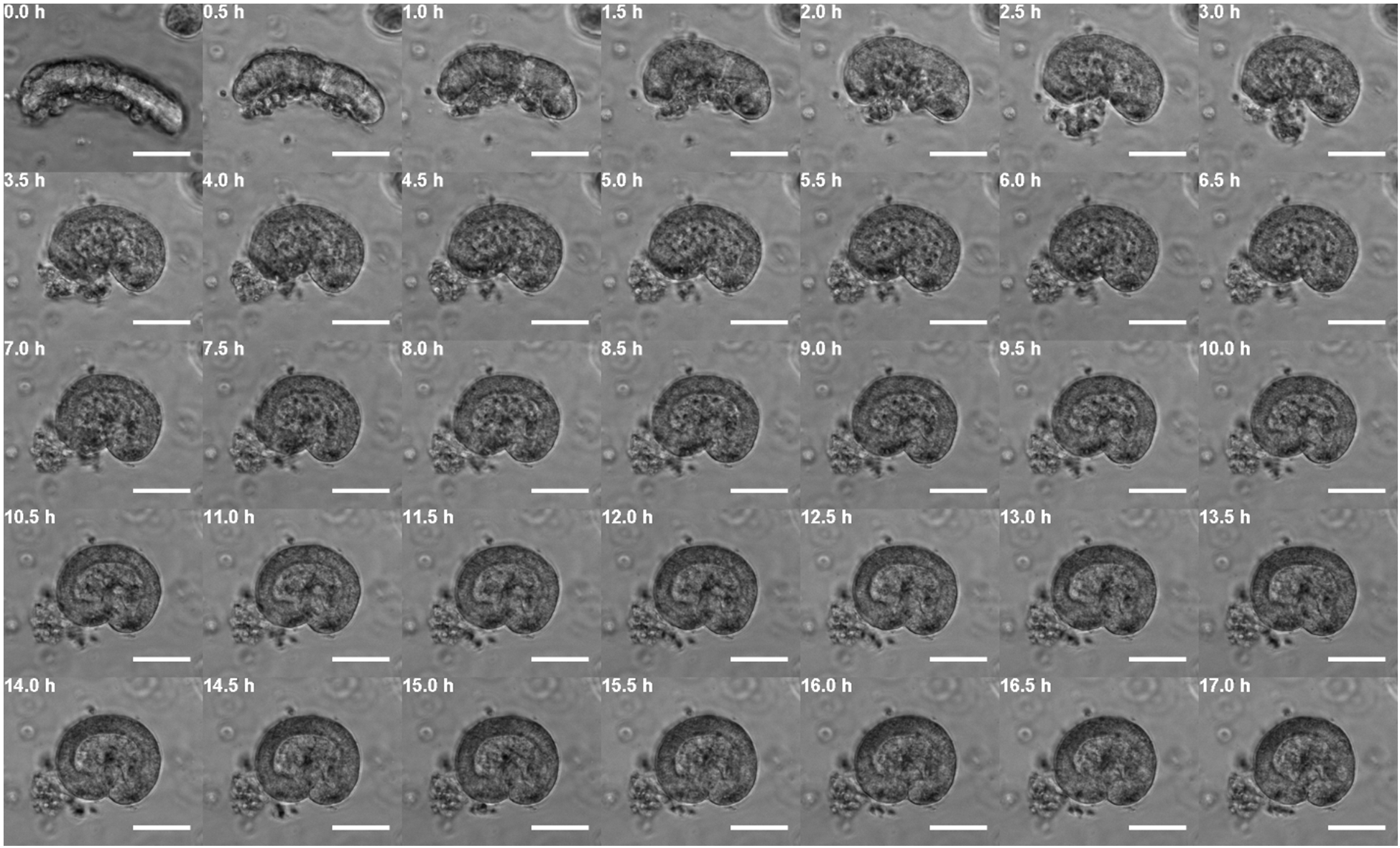

Supplement: Supplementary Figure 1 — Light microscopy time-lapse imaging of a bovine abomasum gland forming an organoid over 17 h after passaging. Scale bar = 50 µm [file Image_1.tif]

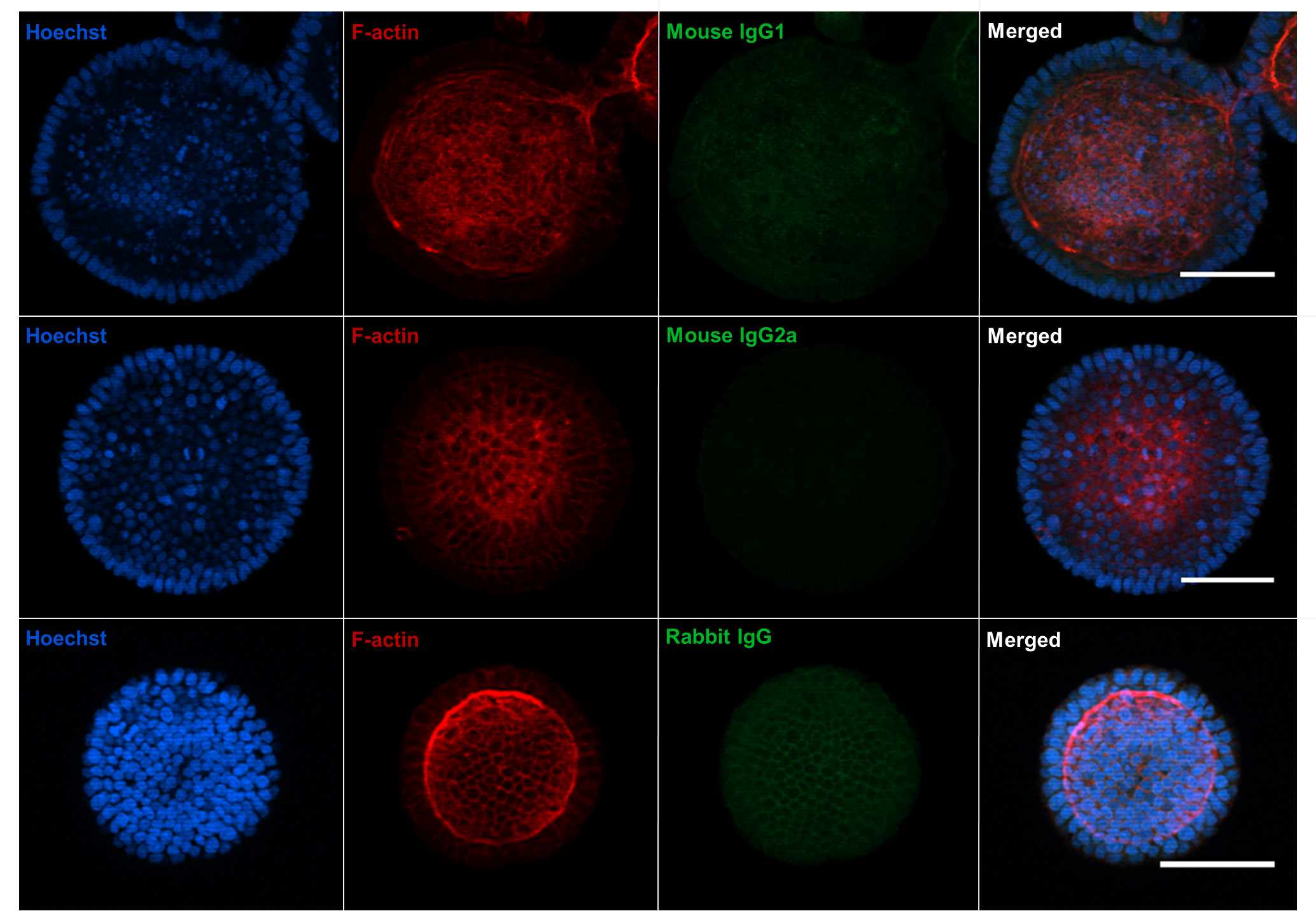

Supplement: Supplementary Figure 2 — Negative controls for immunofluorescence antibody labelling in abomasum organoids ( Figures 2 ). Representative microscopy images of abomasum organoids probed with non-specific host IgG followed by indirect Alexa Fluor® 488-conjugated secondary antibody labelling (green), F-actin (red) and nuclear marker (blue). Labelling control for pan-cytokeratin (top, middle row) and Epcam (bottom row) organoid image represents. Scale bars = 50 µm. [file Image_2.tif]

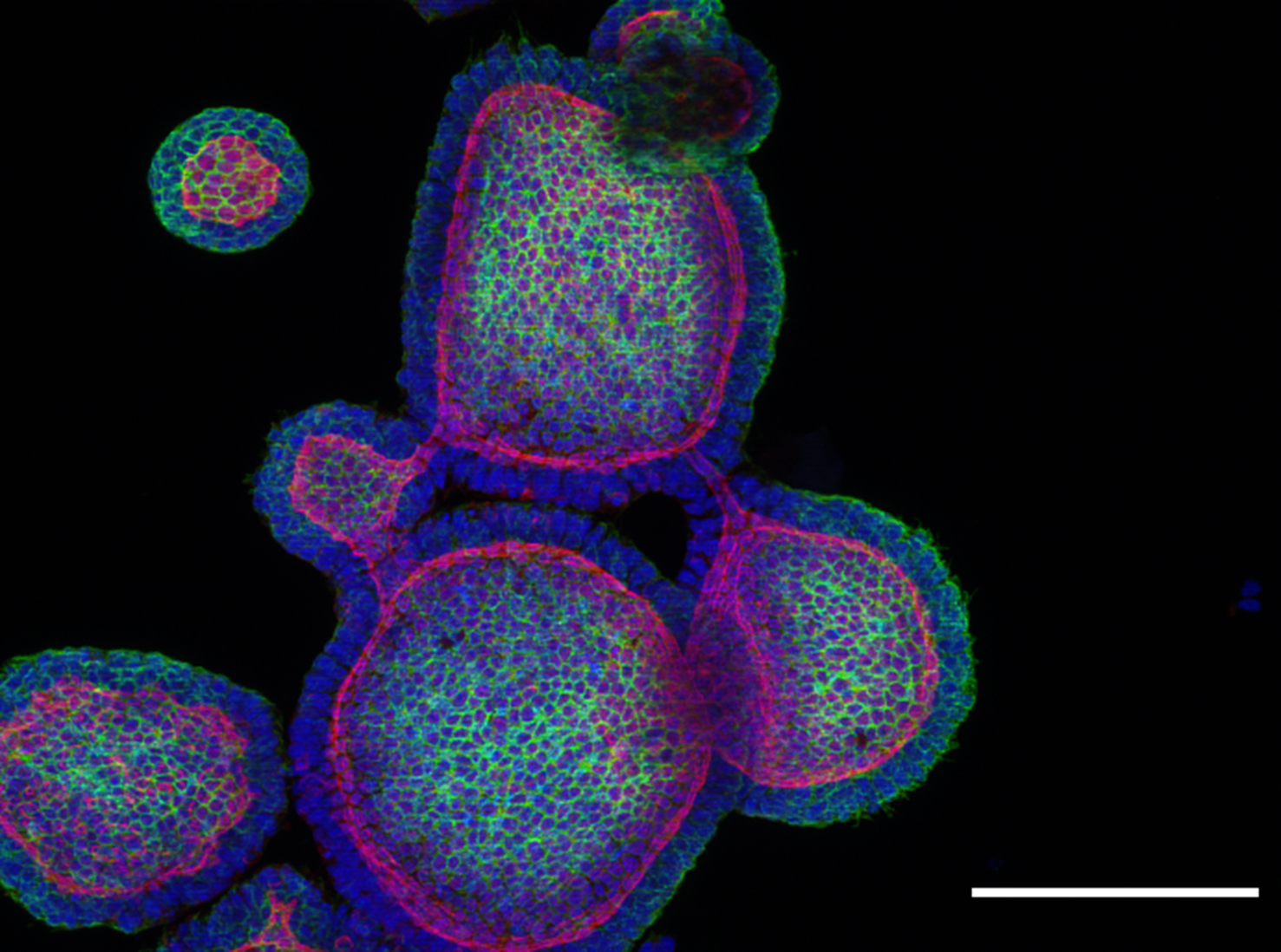

Supplement: Supplementary Figure 3 — Multiple budding abomasal organoids sharing a luminal cavity. Composite and individual channels of a Z-stack orthogonal projection of a representative immunofluorescent image of abomasum oarganoids stained with the epithelial cell markers pan-cytokeratin (green), F-actin (red) and nuclear marker (blue). Scale bar = 100 µm. [file Image_3.tif]

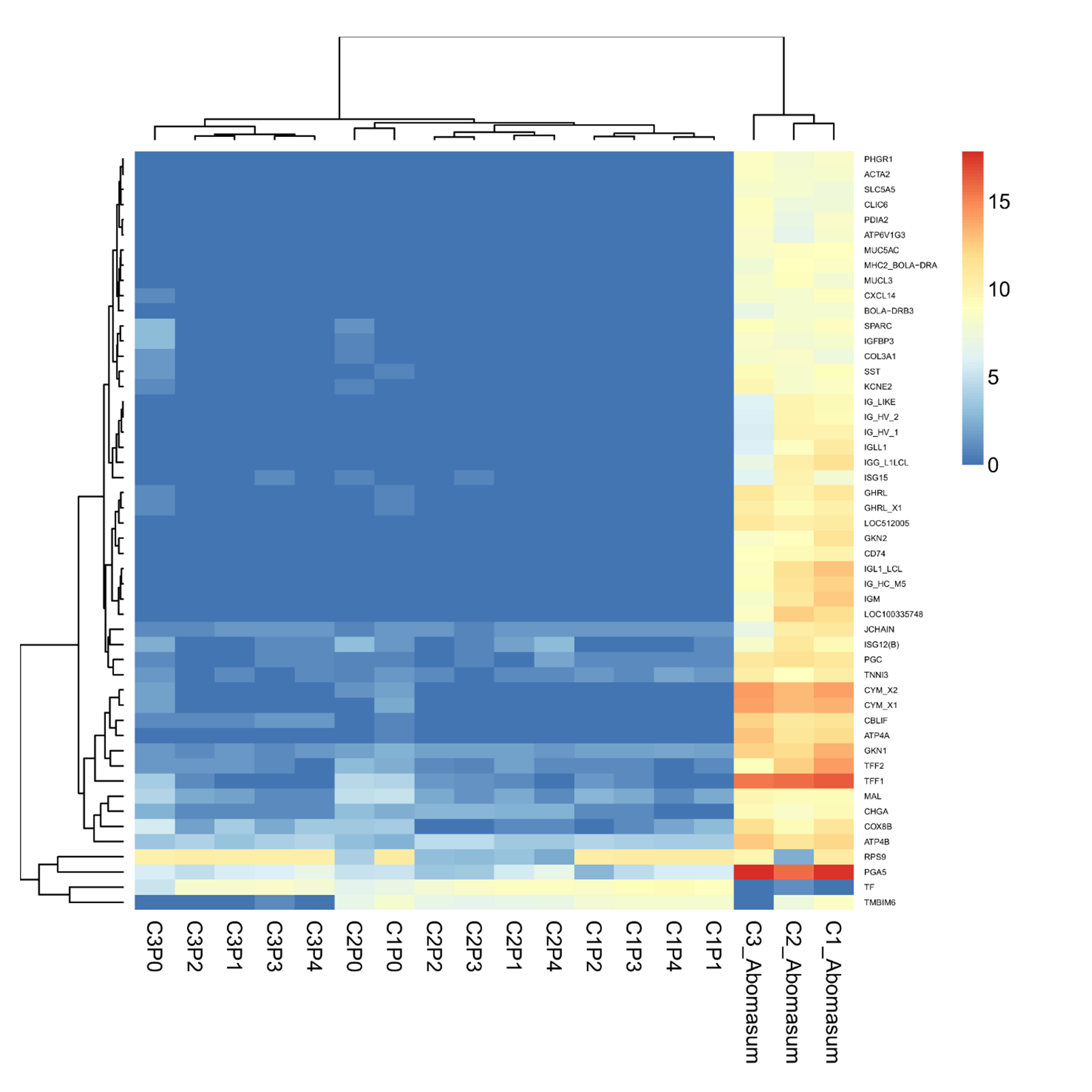

Supplement: Supplementary Figure 4 — Heat map showing expression level of top 50 most variant genes from bovine abomasum and serial passaged abomasum organoids (P0-4). The read count data were normalised using the median of ratios method from the DESeq2 package. Colours indicate level of expression from low (blue) to high (red). The dendrograms indicate similarity between samples and gene expression profiles. Details of genes included in the heat map, are shown in Supplemental File 1 . [file Image_4.tif]

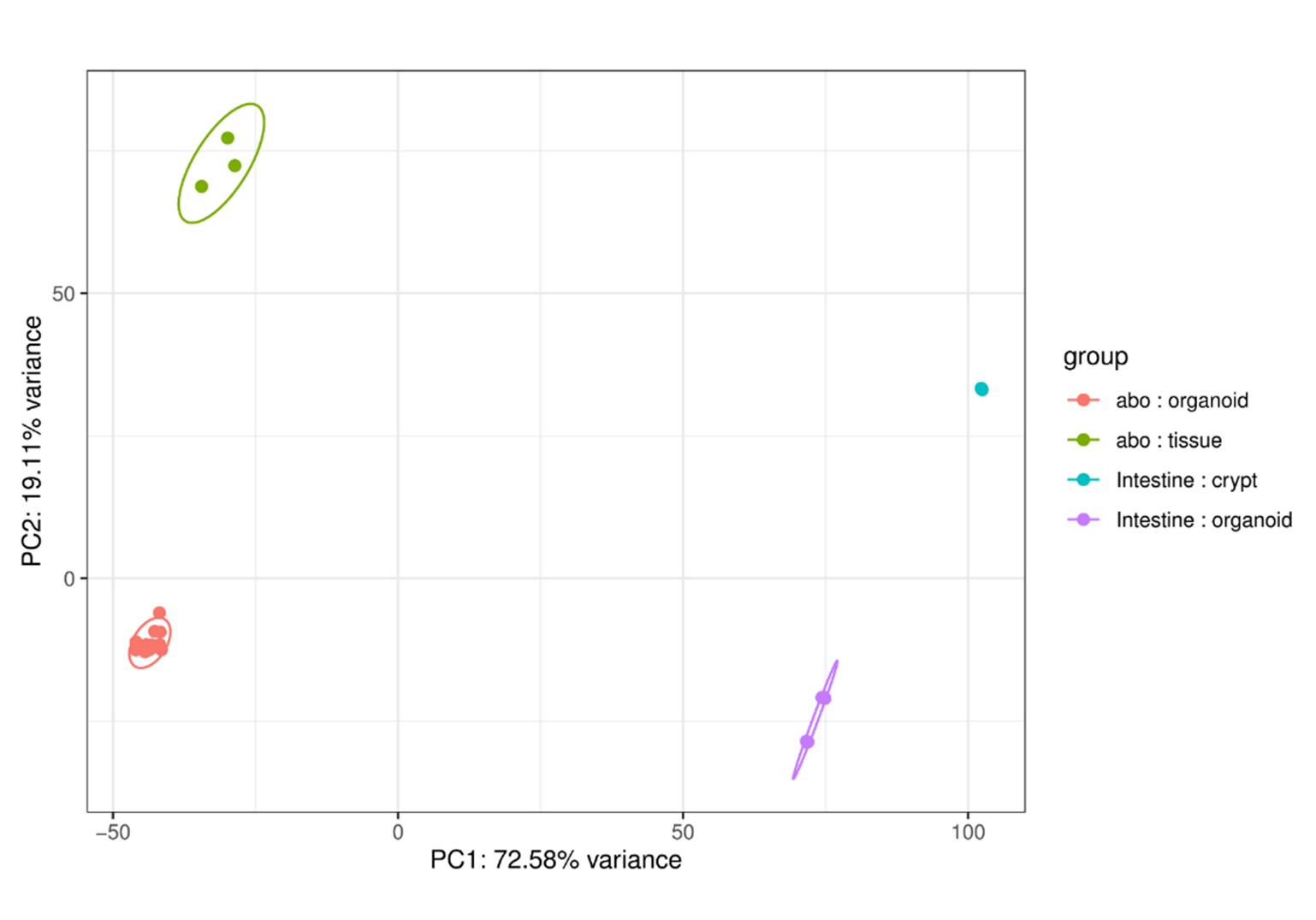

Supplement: Supplementary Figure 5 — Principal component analysis (PCA) of the top 500 most variant genes comparing bovine abomasum and intestine tissue and organoids. The read count data were normalised using the median of ratios method from the DESeq2 package. Sample type is indicated in the key and includes: abomasum organoid (red); abomasum tissue (green); intestinal crypts (blue); intestine organoid (purple). [file Image_5.tif]

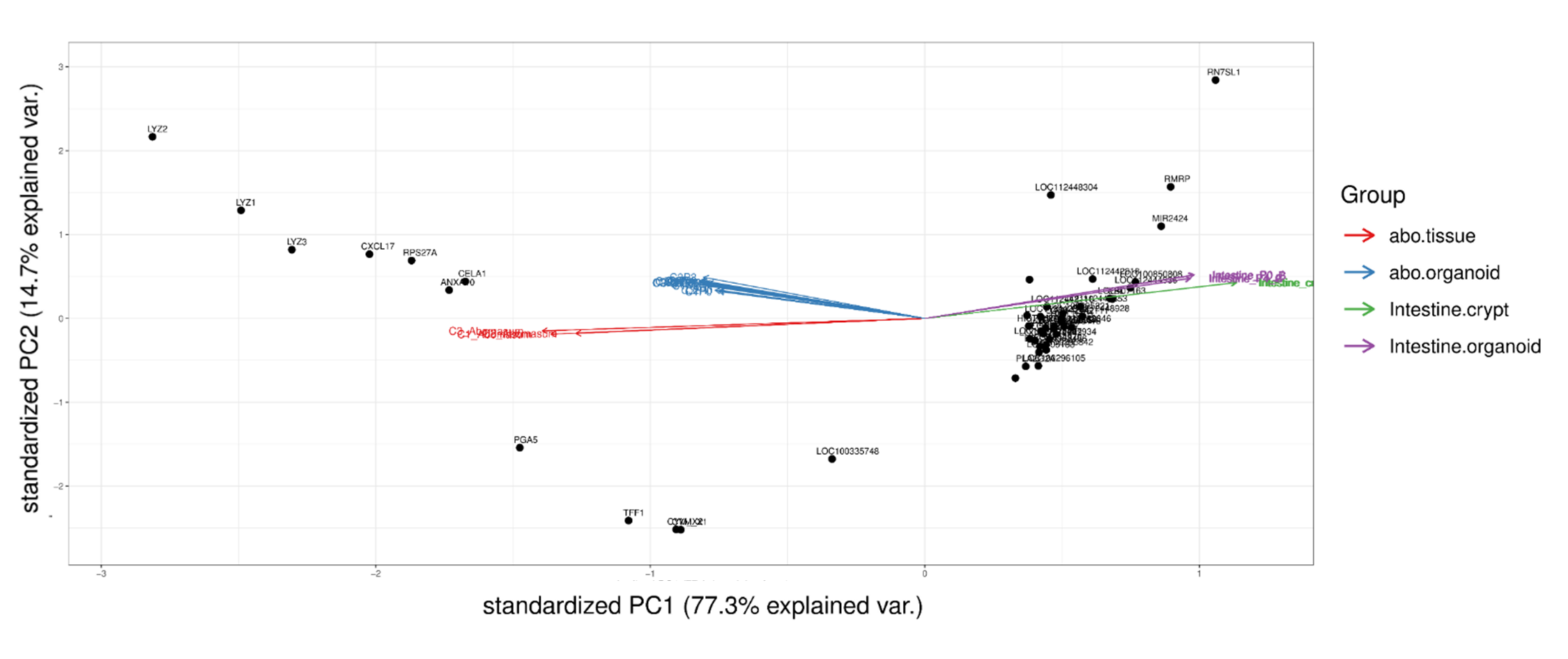

Supplement: Supplementary Figure 6 — Principal component analysis (PCA) of the top 50 most variant genes comparing bovine abomasum and intestine tissue and organoids. The read count data were normalised using the median of ratios method from the DESeq2 package. Sample type is indicated in the key and includes: abomasum organoid (blue); abomasum tissue (red); intestinal crypts (green); intestine organoid (purple). [file Image_6.tif]

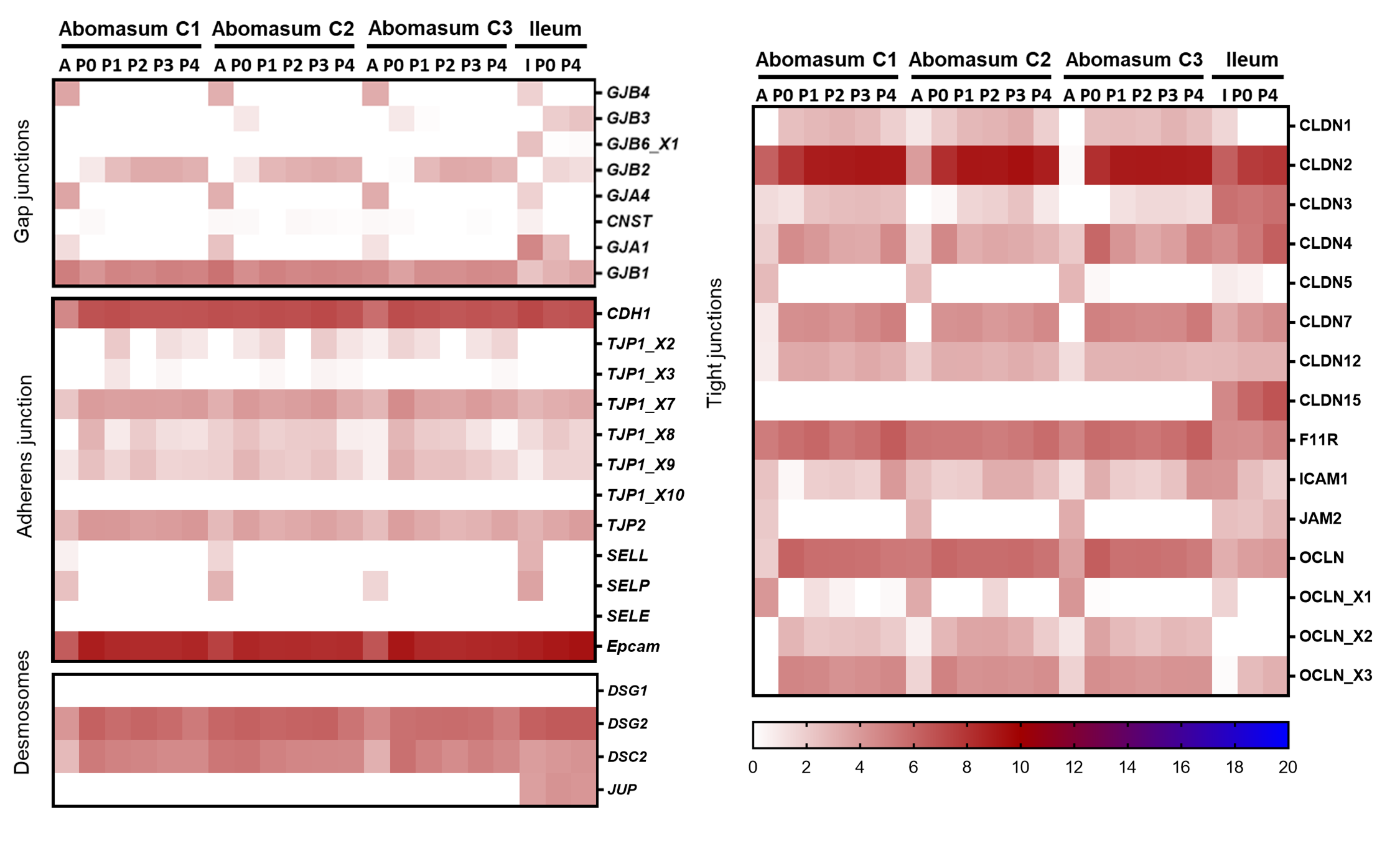

Supplement: Supplementary Figure 7 — Heat map showing the expression of genes associated with cell junctions in abomasum and ileum tissue and organoids. RNA-seq analysis was performed to compare gene expression in abomasal and intestinal tissue respective organoids across multiple passages. Each square from left to right under “abomasum C1-3” and “Ileum” represent cows 1-3 and the pooled intestine samples, primary tissue (A,I) and passages P0-P4. The data was normalised by log2 transformation of transcripts per million reads. Details of genes included in the heat map are shown in Supplemental File 1 . [file Image_7.tif]

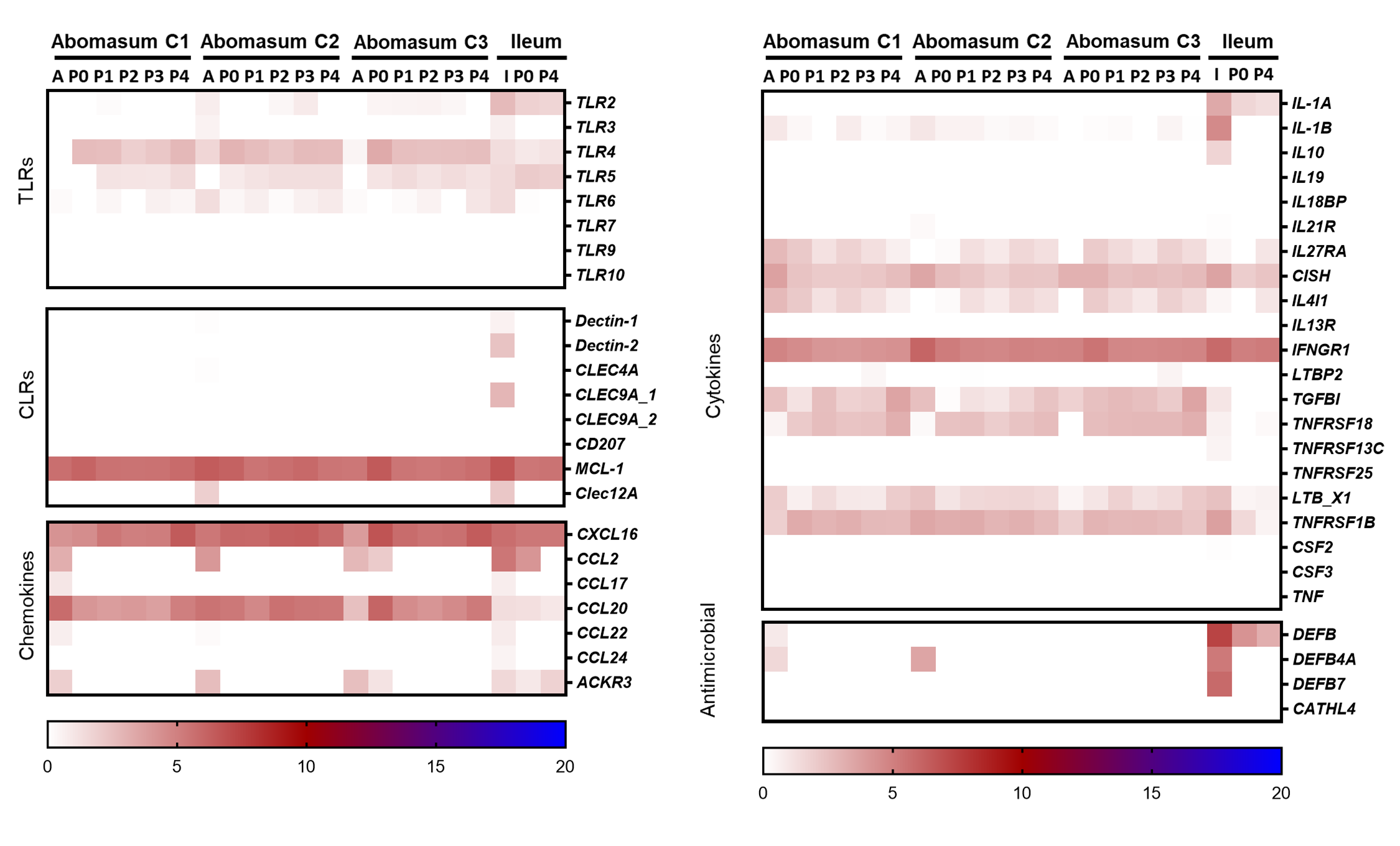

Supplement: Supplementary Figure 8 — Heat map showing the expression of immune-related gene expression in abomasum and ileum tissue and organoids. RNA-seq analysis was performed to compare gene expression in abomasal and intestinal tissue respective organoids across multiple passages. Each square from left to right under “abomasum C1-3” and “Ileum” represent cows 1-3 and the pooled intestine samples, primary tissue (A,I) and passages P0-P4. The data was normalised by log2 transformation of transcripts per million reads. Details of genes included in the heat map are shown in Supplemental File 1 . [file Image_8.tif]

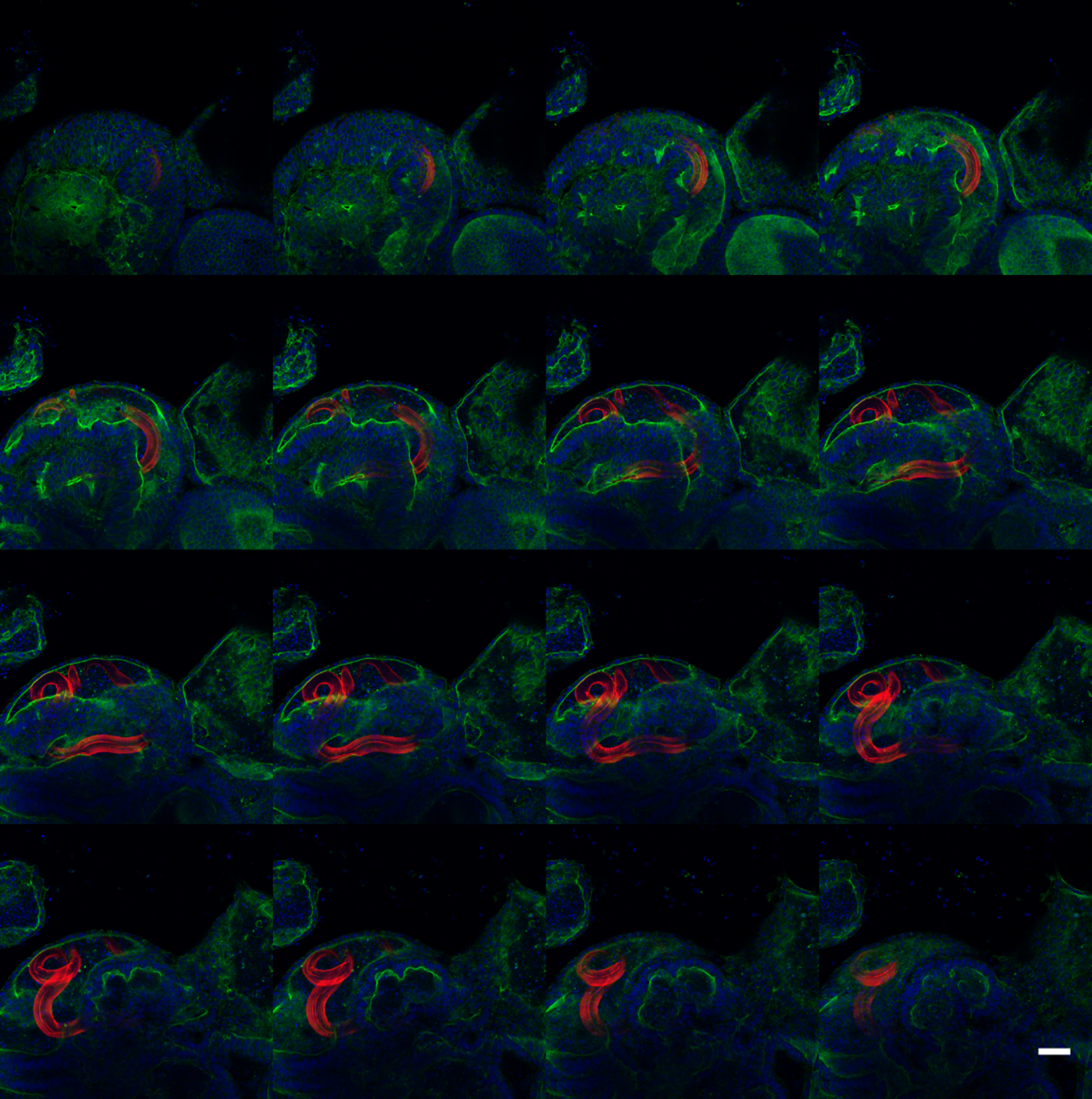

Supplement: Supplementary Figure 9 — Individual Z-stack images of Figures 7C , showing an Ostertagia ostertagi exL3 inside an bovine abomasal organoid. Fluorescent labelling: O. ostertagi exL3 (red), F-actin (green) and nuclear marker (blue). Scale bar = 50 µm, images 1 µm apart. [file Image_9.tif]

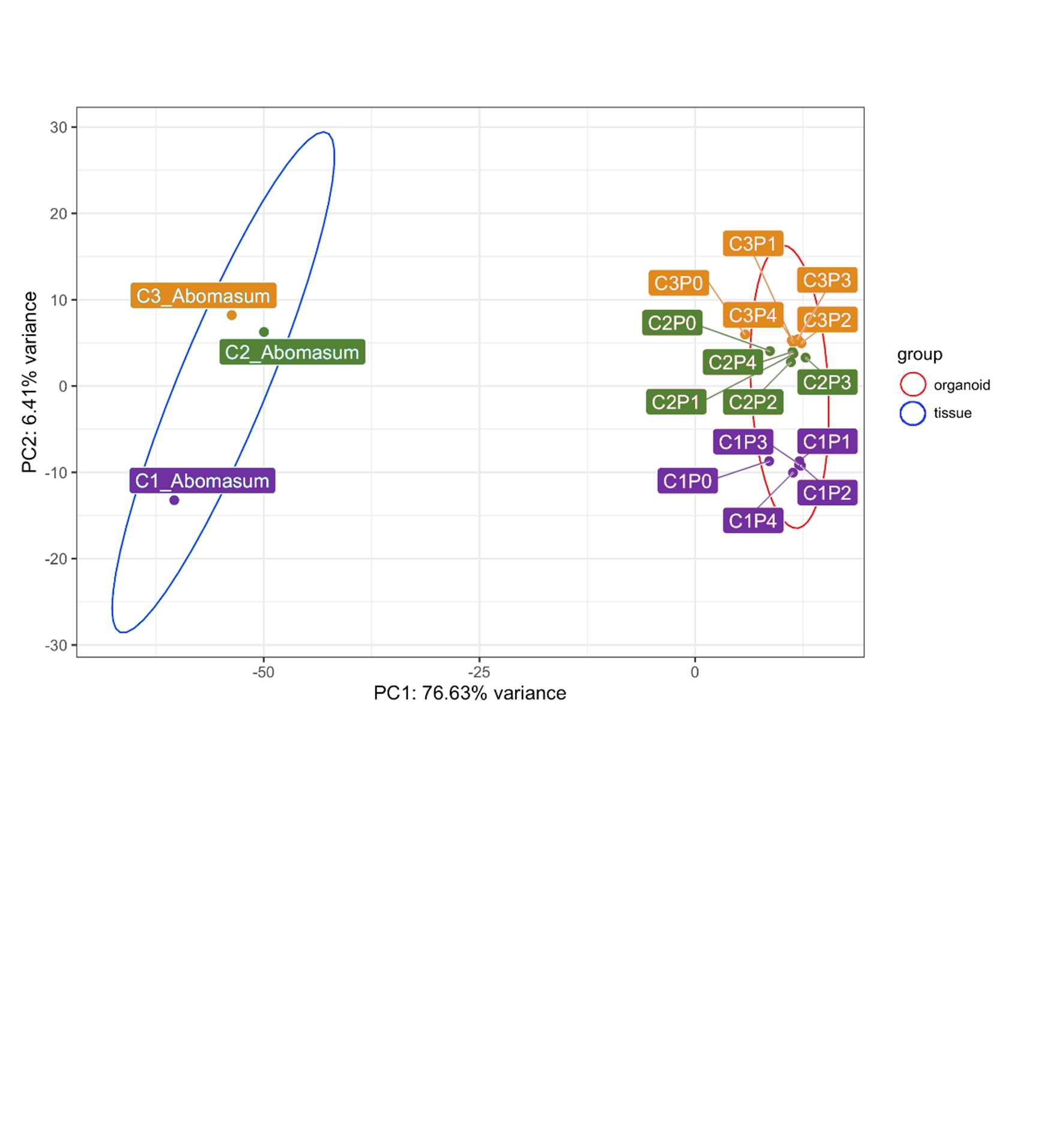

Supplement: Supplementary Figure 10 — Principal component analysis (PCA) of RNA-seq expression of the top 500 most variant genes in bovine abomasum tissue and abomasum organoids from three animals, excluding TMBIM6 and RPSP9. Abomasum tissue and organoids are derived from Calf 1 (Aberdeen Angus; C1, purple), Calf 2 (Holstein-Friesian; C2, green), Calf 3 (Holstein-Friesian; C3, orange). The read count data were normalised using the median of ratios method from the DESeq2 package. Sample type, either tissue or organoid, and organoid passage number (passage 0 – 4; P0 – P4) are indicated in the figure. Ellipses indicates 95% confidence intervals for each cluster. [file Image_10.tif]

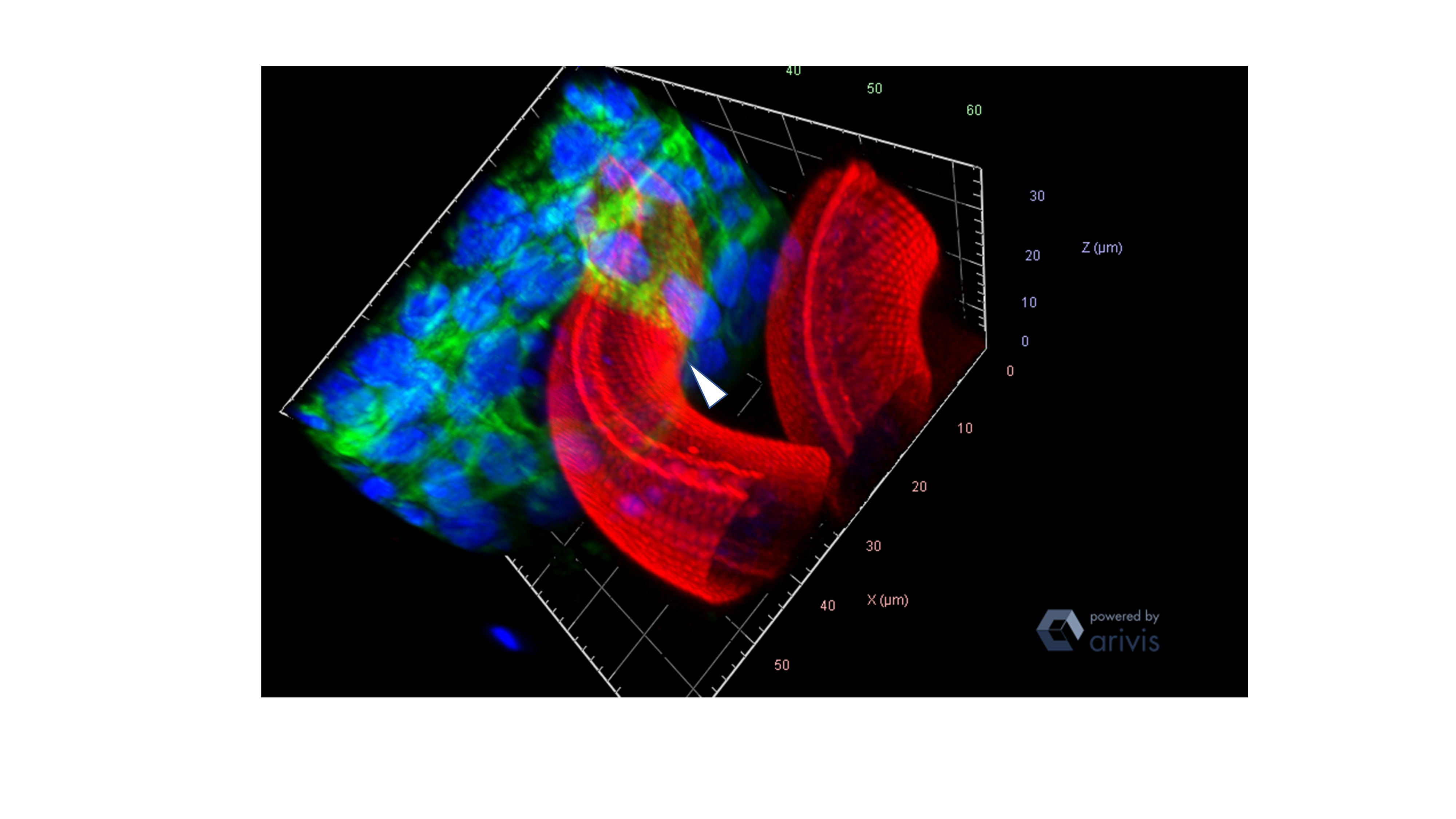

Supplement: Supplementary Figure 11 — 3D representation of the entire Z-stack of Ostertagia ostertagi L3 penetrating the apical surface of the organoid epithelium ( Figures 8 ). Stretched cells and nuclei surrounding the area of exit indicate a paracellular invasion (white arrowhead). Labelling: O. ostertagi exL3 (red), F-actin (green) and nuclear marker (blue). [file Image_11.tif]
